# Chemical Composition and Biological Properties of New Romanian *Lavandula* Species

**DOI:** 10.3390/antiox12122127

**Published:** 2023-12-16

**Authors:** Ionuț Georgică Marchidan, Alina Ortan, Simona Marcu Spinu, Sorin Marius Avramescu, Ionela Avram, Radu Claudiu Fierascu, Narcisa Babeanu

**Affiliations:** 1 Biotechnologies Department, Faculty of Biotechnologies, University of Agronomic Sciences and Veterinary Medicine of Bucharest, 59 Marasti Blvd, District 1, 011464 Bucharest, Romania; ionut.marchidan@biotehnologii.usamv.ro (I.G.M.); narcisa.babeanu@biotehnologii.usamv.ro (N.B.); 2 Mathematics, Physics and Measurements Department, Faculty of Land Reclamation and Environmental Engineering, University of Agronomic Sciences and Veterinary Medicine of Bucharest, 59 Marasti Blvd, District 1, 011464 Bucharest, Romania; simona.spinu@fifim.ro; 3 Department of Inorganic Chemistry, Organic Chemistry, Biochemistry and Catalysis, Faculty of Chemistry, University of Bucharest, 90–92 Soseaua Panduri, 050663 Bucharest, Romania; sorin.avramescu@g.unibuc.ro; 4 Department of Genetics, University of Bucharest, 1-3-Aleea Portocalelor, 060101 Bucharest, Romania; 5 National Institute for Research & Development in Chemistry and Petrochemistry-ICECHIM–Bucharest, 060021 Bucharest, Romania; fierascu.radu@icechim.ro

**Keywords:** Romanian *George 90*, lavender species, chemical composition, promising biological potential

## Abstract

The aims of the present study were to evaluate for the first time the chemical composition and antioxidant, antibacterial, antifungal and antiproliferative potentials of the Romanian *George 90* lavender species, as well as parental species, *L. angustifolia* and *L. latifolia*. The *L. angustifolia*, *L. latifolia* and *George 90* essential oils were analyzed by GC-MS/MS and the *L. angustifolia*, *L. latifolia* and *George 90* hydroalcoholic extracts were analyzed by HPLC-DAD. The antioxidant, antibacterial, antifungal and antiproliferative assays revealed that all the investigated species showed significant activities. The results highlighted the chemical composition and the promising biological potentials of the *L. angustifolia*, *L. latifolia* and *George 90* lavender species, validating their ethnomedicinal value, which offers potential applications as natural drugs.

## 1. Introduction

Antioxidant compounds represent an alternative defense system against reactive oxygen species (ROS) and reactive nitrogen species (RNS), free radicals which can lead to the appearance of various pathologies such as cancer and cardiovascular and neurodegenerative diseases in the case of excessive accumulation [1,2]. When the enzymatic antioxidant mechanism is impaired, antioxidant compounds become an indispensable element to human health. Acquired mostly through diet and various pharmaceutical formulas, such as supplements, they have been used to inhibit as well as prevent oxidative stress [3,4]. Medicinal and aromatic plants, widely used since ancient times in medicine, in cosmetics, in food preservation and in the enhancing of flavors [5], are promising biosources of secondary metabolites with the potential of being developed into novel, effective therapeutic compounds against oxidative stress [6,7].

A prospective source of rich bioactive compounds, the *Lamiaceae* family, is a widespread plant family, comprising numerous medicinal and aromatic plants that provide an array of benefits for human health [8,9,10,11,12]. The *Lavandula* genus of the *Lamiaceae* family contains a multitude of different species with a vast geographical distribution, amongst which the *L. angustifolia* and *L. latifolia* species present the highest economic value [13]. The aforementioned species have found use in the production of soaps, washing agents and perfumes but also in the food and pharmaceutical industries. In addition, lavender also has calming and sedative effects, being therefore used in aromatherapy [3,14].

Multiple studies have investigated the *Lavandula* species and their potential as antioxidant, as well as antitumor and antimicrobial, agents. *L. dentata* has demonstrated effectiveness not only as an antioxidant but also against fungal strains such as *Aspergillus*, *Fusarium* and *Cercospora* and bacterial species ranging from *Streptococcus*, *Pseudomonas* and *Salmonella* to *Listeria monocytogenes*, while silver nanoparticles from aqueous extract showed anticancer potential [15,16]. Mixtures of lavender oil with *C. limon* displayed an effect on *S. epidermidis* [17]. The antimicrobial evaluation of *L. stoechas* essential oil showed noticeable activity against yeast and bacteria, with inhibition zones of 14 mm or more [18] and with an antioxidant activity applicable in food preservation [19]. *L. angustifolia* essential oil vaporization in various areas of a hospital and the monitorization of microbial contamination over a period of three months demonstrated a reduction in the number of bacteria [20]. *L. angustifolia* produced high antioxidant activity, with an inhibition of over 70% measured with the DPPH method [21], and was able to inhibit several *Gram-positive*, *Gram-negative* and yeast strains at small doses [22]. In a study on lung cancer line Calu-3, the vapor phase of the EO of *Lavandula dentata* reached high cytotoxic effects in concentrations of 750 μg/mL by reducing more than 80% of the cell viability in MTT assays and around 60% in SRB assays [23]. Through network pharmacology, lavender essential oil was tested on breast cancer cells and induced apoptosis by the manipulation of the PI3K-AKT pathway [24]. *Lavandula stoechas* essential oil provided antitumor activity against different cancer lines with human gastric AGS cells, reporting over 80% lysis at concentrations of 4 µL/mL (*v*/*v*) [25]. Thus, various lavender species present a high potential as a source of bioactive compounds with beneficial effects in future medicinal developments.

*George 90* is a new lavender species cultivated in southern Romania, obtained by the natural crossing of *L. angustifolia* and *L. latifolia* species. While sharing similarities with its parent species, the new *George 90* distinguishes itself by inflorescence, a calyx and corolla structure, a bract shape and a phytochemical profile. The species has been homologated in Romania since 2017 (Registration certificate no. 4890/07.06.2017).

The purpose of this study was to assess the Romanian lavender species *George 90* in comparison to *L. angustifolia* and *L. latifolia* in regard to the chemical composition and the antioxidant potential of the containing bioactive compounds in order to corroborate its application as a promising therapeutic agent. Different parameters of the extraction method, their effect on the antioxidant yields and the evaluation of the antioxidant activity through complementary assays such as ABTS, DPPH and FRAP are discussed below. HPLC-DAD was used to identify and quantify polyphenolic compounds in hydroalcoholic extracts and GC-MS/MS was used in the analysis of the obtained essential oils. The study also aims to present an analysis of the antibacterial and antifungal activity, with a focus on their effect on *Gram-positive* bacteria and fungi, as well as antiproliferative properties, displayed by the three lavender species.

## 2. Materials and Methods

### 2.1. Sampling and Preparation Steps of Plant Materials

The sampling of plant material was carried out from two nurseries located in Buftea, Ilfov, Romania and Horodnic de Sus, Suceava, Romania. Based on the data presented in the specialized literature and the periodic preliminary studies regarding the content of compounds present in the essential oils and extracts, sampling was carried out in June 2022, when the studied lavender species (*L. angustifolia*, *L. latifolia* and *George 90*) reached maturity (flowering percentage over 50%, full and robust flower spikes and woody lower stems) [21]. The process of sampling the plant material was carried out in polyethylene bags that were disposable, dark and hermetically sealed, and its transport was carried out in refrigerated crates at a temperature of 4 °C. The plant material was selected in the laboratory and separated from other plant residues, root and sediment. The inflorescences were separated from the rest of the aerial parts of the plant and used to extract the essential oil. The aerial parts were dried in an oven at 40 °C until a constant mass was obtained and then used for the extraction of polyphenolic compounds.

### 2.2. Extraction Steps of the Essential Oils

#### 2.2.1. Microwave-Assisted Extraction

The microwave-assisted extraction (MAE) of the essential oil was performed using an advanced Milestone ETHOS X (Milestone, Sorisole, Italy) microwave extraction system. The system features a Clevenger-type glass installation, which is attached to the top of the furnace, having as a principle the continuous condensation of compounds [26]. The essential oil was extracted from lavender inflorescences (*L. angustifolia*, *L. latifolia* and *George 90*). In total, three MAE essential oils were obtained: *L. angustifolia* MAE essential oil, *L. latifolia* MAE essential oil and *George 90* MAE essential oil. The MAE extraction of the essential oils was carried out at atmospheric pressure, using a heat-resistant glass reactor with a capacity of 5 L, closed with a heat-resistant glass lid, at a power of 1000 W, for 120 min. Prior to the extraction process, 450 g of dried lavender inflorescence biomass underwent a 30 min moistening phase within a vessel containing 2 L of ultrapure water at a biomass:water ratio of 1:45 (*w*/*v*); subsequently, the well-drained vegetal material was introduced into the glass reactor for essential oil extraction.

#### 2.2.2. Hydrodistillation

In order to compare the extraction efficiency of the MAE extraction, the extraction of the essential oil by hydrodistillation (HD) at atmospheric pressure was carried out in parallel [27]. In total, 450 g of lavender inflorescences (*L. angustifolia*, *L. latifolia* and *George 90*) was placed in a 3 L Erlenmeyer flask equipped with a heating hob and a Clevenger glass installation (Behr Labor-Technik GmbH, Düsseldorf, Germany), into which 2 L of ultrapure water in a biomass:water ratio of 1:45 (*m*/*v*) was introduced. The HD extraction of the essential oils was performed for 240 min. In total, three HD essential oils were obtained by HD extraction: *L. angustifolia* HD essential oil, *L. latifolia* HD essential oil and *George 90* HD essential oil. After each extraction process, both MAE and HD extractions, each essential oil was separated from the aqueous phase and collected in brown vials, sealed with polytetrafluoroethylene caps and then stored at 4 °C. The essential oil content was expressed in % (*w*/*w*), and the extraction yield of the essential oil (%) was calculated as follows:

(1) $$Extraction\;yield\;\left(\%\right)=\frac{essential\;oil\;(g)}{lavender\;inflorescences\;(g)}*100$$

### 2.3. Extraction Steps of Polyphenolic Compounds

The extraction of polyphenolic compounds was carried out using an advanced MAE technique and the microwave extraction system Milestone ETHOS X (Milestone, Sorisole, Italy) [28]. The influence of the solvent (ethanol and methanol) and the particle size of the plant material on the quality and quantity of polyphenolic compounds was tested. Extraction was performed on the aerial parts of *L. angustifolia, L. latifolia* and *George 90*, respectively. The dried plant material was ground to obtain a coarse-sized homogeneous powder (350 rpm for 10 s) and a very small homogeneous powder (350 rpm for 50 s) using the Grindomix GM 200 knife mill (Retsch, Duesseldorf, Germany). For each experiment, 1.5 g of plant material (coarse and fine-grounded, respectively) was mixed with ethanol, in concentrations of 50%, and 70% respectively, at a plant:solvent ratio of 1:20 (*w*/*v*) and 1:40 (*w*/*v*), respectively, resulting in eight hydroalcoholic extracts in total. The same experiments were performed using methanol as a solvent, in a concentration of 50%, and at a plant:solvent ratio of 1:20 (*w*/*v*) and 1:40 (*w*/*v*), respectively, resulting in four hydroalcoholic extracts in total. The same experiments were performed using methanol as a solvent, in a concentration of 50%, and at a plant:solvent ratio of 1:20 (*w*/*v*) and 1:40 (*w*/*v*), respectively, resulting in four hydroalcoholic extracts in total. The extraction parameters were: temperature—70 °C, time—60 min and microwave power—500 W. The obtained extracts underwent vacuum filtration with Whatman No. 1 filter paper, followed by concentration using a nitrogen stream concentrator (Biobase Group, Jinan, Shandong, China) at 40 °C. Subsequently, the concentrates were freeze-dried using the Alpha 3 lyophilizer (LSCbasic, Hristos, Osterode am Harz, Germany). In total, 12 different extracts for each lavender species (*L. angustifolia*, *L. latifolia* and *George 90*) were obtained by MAE extraction. Each extraction was carried out in triplicate. The 108 extracts obtained were stored in brown glass vials at −18 °C.

The polyphenolic compound content was expressed in % (*w*/*w*) and the extraction yield of polyphenolic compounds (%) was calculated as follows:

(2) $$Extraction\;yield\;\left(\%\right)=\frac{lavander\;extract\;(g)}{lavander\;aerial\;parts\;(g)}*100$$

### 2.4. GC-MS/MS Analysis of Essential Oils

The analysis of *L. angustifolia*, *L. latifolia* and *George 90* essential oils was carried out using the system GC-MS/MS TSQ 8000 Evo Triple Quadrupole (Thermo Fisher Scientific Inc., Waltham, MA, USA), equipped with a capillary column TG-5SILMS (30 m × 0.25 mm × 0.25 µm). The *L. angustifolia*, *L. latifolia* and *George 90* essential oils obtained by MAE extraction (LA-MAE, LL-MAE and G90-MAE) and the *L. angustifolia*, *L. latifolia* and *George 90* essential oils obtained by classical hydrodistillation (LA-HD, LL-HD and G90-HD) were analyzed. The separation of compounds employed the following oven temperature program: an initial temperature of 40 °C was maintained for 5 min, followed by a gradual increase at a rate of 5 °C/min until reaching 250 °C, where it was held for 1 min. An essential oil concentration of 3% (isooctane) was used, and the injection port was set at 250 °C (injection volume 1 μL at a split ratio of 1:150). The ion source and interface were maintained at 280 °C and 300 °C. Helium was used as the carrier gas at a constant 1.0 mL/min flow rate. The mass spectrometer was operated at a scan interval between 35 and 250 *m*/*z*, and the processing of the obtained data was carried out with the help of the Thermo Xcalibur 3.0 program.

### 2.5. HPLC-DAD Analysis of Polyphenolic Compounds

The L-3000 High-Performance Liquid Chromatography system (Rigol Technologies, INC Beijing, Beijing, China) was used for the separation, identification and quantification of polyphenolic compounds present in the *L. angustifolia*, *L. latifolia* and *George 90* hydroalcoholic extracts. In the HPLC-DAD analysis, a Phenomenex C18 column (150 mm × 4.6 mm, 5 µm particle size) and an injection volume of 10 µL were used. The column oven temperature was set at 35 °C and the mobile phases used were (A) 0.1% trifluoroacetic acid (TFA) in water and (B) 0.1% TFA in acetonitrile. The elution gradient was 0–100% B for 60 min and the elution flow rate was set at 0.8 mL/min. A total of 21 reference compounds from the polyphenolic class were used, such as: tannic acid, gallic acid, protocatechuic acid, catechin, caffeic acid, chlorogenic acid, syringic acid, epicatechin, p-coumaric acid, ferulic acid, o-coumaric, ellagic acid, isoquercetin, rutin, rosmarinic acid, hyperoside, naringin, quercetin, luteolin, naringenin and kaempferol. For the detection of polyphenolic compounds, four different fixed λ wavelengths (255 nm, 280 nm, 325 nm and 355 nm) were used, according to the λ_max_ obtained when tracing the molecular absorption spectra, from the λ range of 200–400 nm. The identification and quantification of the polyphenolic compounds were achieved by comparison with the spectra of the reference compounds, at each retention time. The stock solutions of the 21 reference compounds were prepared at a concentration of 6 mg/mL, and different concentrations in the linear range 0.98–250 µg/mL were used for the calibration curves. For each *L. angustifolia*, *L. latifolia* and *George 90* hydroalcoholic extract, the chromatographic analysis was performed in triplicate.

### 2.6. Antioxidant Potential of Bioactive Compounds

#### 2.6.1. Evaluation of Antioxidant Potential by Inhibiting the DPPH Free Radical

Antioxidant potential was determined by the DPPH method [29], adapted for microplate reader spectrophotometry, using a UV–Vis spectrophotometer (Tecan Group Ltd., Männedorf, Switzerland). Briefly, equal volumes of 250 µM DPPH solution in ethanol and lavender samples (*L. angustifolia*, *L. latifolia* and *George 90* essential oils and hydroalcoholic extracts) were added to the microplate. The samples were homogenized and incubated in darkness at room temperature for 30 min, and the absorbance of lavender samples was read at a λ of 517 nm. Trolox, the antioxidant reference compound, was used to determine antioxidant activity equivalents, namely, micrograms of trolox per milliliter of essential oil (μgEqT/mL) and milligrams of Trolox per gram of the plant (mgEqT/g). The tests were performed in three independent measurements (*n* = 3), and the inhibition percent of the DPPH free radical of the lavender samples and of the reference compound, Trolox, was calculated using the equation:

(3) % inhibition=[(Abs mc−Abs mp)/Abs mc]∗100

where m_c_ represents the average of the absorbance values of the control samples, and m_p_ represents the average of the absorbance values of the lavender samples.

#### 2.6.2. Evaluation of Antioxidant Potential by Inhibiting the ABTS^●+^ Cationic Radical

The ABTS assay was performed using the Busuioc et al. methodology [30], adapted for microplate reader spectrophotometry, and using a UV–Vis spectrophotometer (Tecan Group Ltd., Männedorf, Switzerland). First, ABTS^●+^ radical cation stock solution was prepared by mixing equal amounts of 7.8 mM 2,2′-azino-bis(3-ethylbenothiazoline-6-sulfonic acid) ammonium salt solution and 140 mM potassium persulfate solution. The mixture was incubated to react for 12 h in the darkness at room temperature. The solution was then diluted in methanol to obtain an absorbance of 0.02 ± 1.1 units at a λ of 734 nm. Equal volumes of ABTS^●+^ solution and lavender samples (*L. angustifolia*, *L. latifolia* and *George 90* essential oils and hydroalcoholic extracts) were added to the microplate. The samples were homogenized and incubated in the darkness at room temperature for 30 min, and the absorbance of lavender samples was read at a λ of 734 nm. Trolox, the antioxidant reference compound, was used to determine antioxidant activity equivalents, namely, micrograms of trolox per milliliter of essential oil (μgEqT/mL) and milligrams of trolox per gram of the plant (mgEqT/g). The tests were performed in three independent measurements (*n* = 3), and the inhibition percent of the ABTS^●+^ cation radical of the lavender samples and of the reference compound, Trolox, was calculated using the equation:

(4) % inhibition=[(Abs mc−Abs mp)/Abs mc]∗100

where m_c_ represents the average of the absorbance values of the control samples, and m_p_ represents the average of the absorbance values of the lavender samples.

#### 2.6.3. Evaluation of Antioxidant Potential by Ferric Ion Reduction (FRAP)

Antioxidant potential was determined by the FRAP method [31], adapted for microplate reader spectrophotometry, using a UV–Vis spectrophotometer (Tecan Group Ltd., Männedorf, Switzerland). Briefly, the FRAP solution was prepared by mixing 100 mL of acetate buffer solution (300 mM, pH 3.6), 10 mL of 2,4,6-tris (2-pyridyl)-s-triazine (TPTZ) solution (10 mM in 40 mM HCl) and 10 mL of FeCl_3_ (20 mM). The FRAP solution and lavender samples (*L. angustifolia*, *L. latifolia* and *George 90* essential oils and hydroalcoholic extracts) were added to the microplate. The samples were homogenized and incubated in the darkness at room temperature for 30 min, and the absorbance of the lavender samples was read at a λ of 593 nm. Trolox, the antioxidant reference compound, was used to determine antioxidant activity equivalents, namely, micrograms of trolox per milliliter of essential oil (μgEqT/mL) and milligrams of trolox per gram of the plant (mgEqT/g). The tests were performed in three independent measurements (*n* = 3).

### 2.7. Evaluation of the Antimicrobial Potential of Bioactive Compounds against Gram-Positive Bacteria

#### 2.7.1. Minimum Inhibitory Concentration

The minimum inhibitory concentration (MIC) of the *L. angustifolia*, *L. latifolia* and *George 90* essential oils was evaluated by the microdilution method [32,33]. Different concentrations of essential oils (between 0.31% and 10%) were tested, using Mueller Hinton Broth (MHB) as the culture medium. *Gram-positive* bacterial strains, namely, *Bacillus cereus* and *Bacillus subtilis*, were used to obtain an individual bacterial suspension of three to five colonies suspended in 9 mL of sterile distilled water. An aliquot of bacterial cells (50 μL) was added to microplates, along with *L. angustifolia*, *L. latifolia* and *George 90* essential oils, serially diluted in MHB. The microplates were incubated at 35 °C for 18–24 h, and the obtained results were evaluated macroscopically. Experiments were performed in duplicate, and the mean MIC value was calculated. Positive control (no sample) and negative control (no inoculum) samples were performed for each experiment. Ciprofloxacin was used as a standard antibacterial drug.

#### 2.7.2. Minimum Bactericidal Concentration

The minimum bactericidal concentration (MBC) was assessed for bacterial suspensions in wells with concentrations equal to the MIC value, above the MIC and below the MIC of *L. angustifolia*, *L. latifolia* and *George 90* essential oils [34]. For this purpose, after 24 h of incubation of the bacterial cultures, treated with *L. angustifolia*, *L. latifolia* and *George 90* essential oils, 10 μL of each bacterial suspension was taken and distributed on the surface of a plate with Mueller Hinton Agar (MHA). The plates were incubated for 18–24 h, at a temperature of 35 °C, and the results obtained were evaluated macroscopically. The experiments were performed in duplicate and the mean MBC value was calculated. Positive control (no sample) and negative control (no inoculum) samples were performed for each experiment. Ciprofloxacin was used as a standard antibacterial drug.

### 2.8. Evaluation of the Antimicrobial Potential of Bioactive Compounds against Different Types of Fungus

#### 2.8.1. Minimum Inhibitory Concentration

The minimum inhibitory concentration (MIC) of the *L. angustifolia*, *L. latifolia* and *George 90* essential oils was evaluated by the microdilution method [35,36]. Different concentrations of essential oils (between 1.25% and 40%) were tested, using Potato Dextrose Broth (PDB) as a culture medium. The fungal species *Aspergillus brasiliensis*, *Fusarium oxysporum* and *Penicillium expansum* were used to obtain an individual spore suspension of three to five colonies suspended in 9 mL of sterile distilled water. An aliquot of fungal cells (50 μL) was added to microplates, along with *L. angustifolia*, *L. latifolia* and *George 90* essential oils, serially diluted in PDB. The microplates were incubated at 25 °C for seven days and the results obtained were evaluated macroscopically. The experiments were performed in duplicate and the mean MIC value was calculated. Positive control (no sample) and negative control (no inoculum) samples were performed for each experiment. Fluconazole was used as a standard antifungal drug.

#### 2.8.2. Minimum Fungicide Concentration

The minimum fungicidal concentration (MBC) was assessed for fungal suspensions in wells with concentrations equal to the MIC value, above the MIC and below the MIC of *L. angustifolia*, *L. latifolia* and *George 90* essential oils [34]. For this purpose, after the seven days of incubation of the fungal species *Aspergillus brasiliensis*, *Fusarium oxysporum* and *Penicillium expansum*, treated with *L. angustifolia*, *L. latifolia* and *George 90* essential oils, 10 μL of each fungal suspension was taken and distributed on the surface of a plate with Potato Dextrose Agar (PDA). The plates were incubated for seven days at 25 °C and the results obtained were evaluated macroscopically. The experiments were performed in duplicate and the mean MFC value was calculated. Positive control (no sample) and negative control (no inoculum) samples were performed for each experiment. Fluconazole was used as a standard antifungal drug.

### 2.9. Evaluation of the Antiproliferative Potential of Bioactive Compounds

Human cervix carcinoma cells (HeLa) were selected for the antiproliferative potential assessment of the *L. angustifolia*, *L. latifolia* and *George 90* essential oils using the MTT assay. Two different dilutions (1 and 10%) of the essential oils were analyzed. PBS was used as a negative control. HeLa cells were cultivated in an RPMI-1640 culture medium (Sigma-Aldrich, St. Louis, MO, USA), supplemented with 2 mM Glutamine (Sigma-Aldrich), 10% heat-inactivated Fetal Bovine Serum (FBS) (Sigma-Aldrich) and 1% Pen/Strep (penicillin/streptomycin solution, 50 µg/mL—Sigma-Aldrich) for 24 h at 37 °C and 95% humidity with 5% CO_2_. After 24 h, the supernatant was removed, the cells were washed with PBS (Phosphate Buffered Solution—Sigma-Aldrich), MTT (0.5 mg/mL DMSO, Sigma, St. Louis, MO, USA) was added and the plates were incubated for 4 h at 37 °C. The purple formazan formed was dissolved in 200 μL DMSO. The optical density was measured at 570 nm using a microplate reader (Synergy™ HTX Multi-Mode Microplate Reader, Biotek, Winooski, VT, USA).

### 2.10. Statistical Analysis

The experiments were performed in triplicate, and the data presented represent the average of the three determinations ± standard deviation (SD). The statistical evaluation of the obtained data was assessed by one-way analysis of variance (ANOVA), followed by the TUKEY test, to find out significant differences (*p* < 0.05). The results from the antiproliferative assay were represented using the GraphPad Prism 9 software (San Diego, CA, USA).

## 3. Results

### 3.1. Sampling and Preparation of Plant Materials

**Figure 1:**
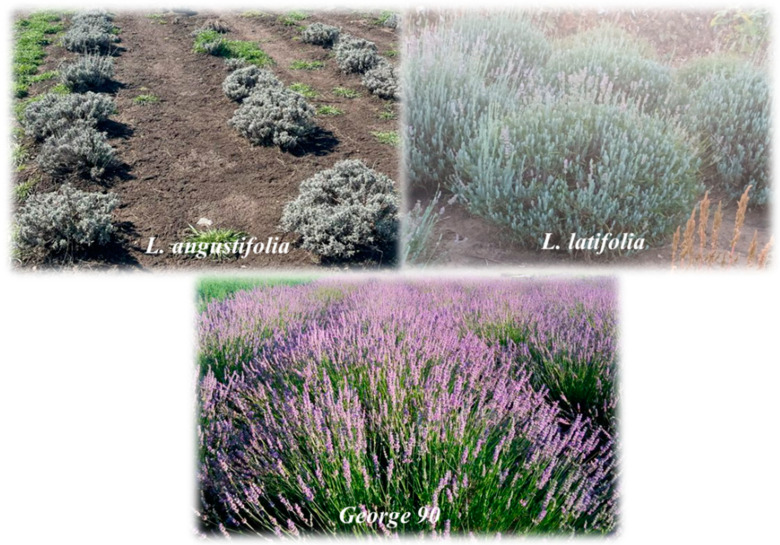
Images from the sampling site of the studied lavender species. *L. angustifolia*—Horodnic de Sus nursery, *L. latifolia* and *George 90*—Buftea nursery.

The geographical and sampling areas of the lavender species are of significant importance to the ecology and biology of the plant [37]. In the initial phase of the experimental study, *L. angustifolia*, *L. latifolia* and *George 90* plant materials (Figure 1) were collected from two nurseries. Specifically, *L. angustifolia* (LA) plant material was harvested from the Buftea, Ilfov County nursery, while *L. latifolia* (LL) and *George 90* (G90) plant materials were harvested from the Horodnic de Sus, Suceava County nursery. The sampling process, an important step in achieving the research objectives, was carried out in June 2022, when the lavender species reached maturity and the bioactive compounds reached their maximum concentration. Following the sampling phase, the plant material underwent preparation and subsequent analysis stages.

### 3.2. Essential Oils Extraction

The most common method of essential oil extraction is hydrodistillation (HD), but this requires an extended extraction time. Additionally, at the temperature of the hydrodistillation process, close to 100 °C, the polymerization of aldehydes takes place, and esters could be partially degraded by hydrolysis, resulting in acids and alcohols. Microwave-assisted extraction (MAE) combines microwave extraction and conventional hydrodistillation extraction, offering multiple advantages over the conventional method [38].

In the current research study, advanced MAE extraction was used to obtain lavender essential oil rich in bioactive compounds. Conventional HD extraction was performed as a comparative method. Lavender essential oil was obtained from the inflorescences of three different lavender species, such as *L. angustifolia*, *L. latifolia* and *George 90*, respectively. In total, six essential oils were obtained: *L. angustifolia* MAE essential oil (LA-MAE), *L. latifolia* MAE essential oil (LL-MAE), *George 90* MAE essential oil (G90-MAE), *L. angustifolia* HD essential oil (LA-HD), *L. latifolia* HD essential oil (LL-HD) and *George 90* HD essential oil (G90-HD), respectively. The extraction yield of the essential oils extracted from the LA, LL and G90 lavender species is presented in Table 1. The results indicated a significantly greater yield of essential oil through the MAE extraction method compared to the extraction achieved through HD extraction. The highest extraction yield was obtained for the essential oil extracted from G90 lavender species, by both the MAE and HD extraction methods.

### 3.3. Polyphenolic Compounds Extraction

The microwave-assisted extraction (MAE) method was used to obtain extracts rich in polyphenolic compounds from the aerial parts of LA, LL and G90 lavender species, using polar solvents, ethanol (EtOH) and methanol (MeOH), in order to obtain a higher extraction yield. MAE extraction involves the instantaneous heating of the system, which regulates the heat transfer during the extraction process and thus improves the extraction yield.

The temperature (70 °C) was chosen considering the boiling points of the two solvents (ethanol and methanol). The temperature depends on the relative dielectric properties of the solvent and the sample, respectively, which change during the extraction process. The presence of water alongside the solvent (EtOH or MeOH) enhances the efficiency of heating within the vessel, facilitating the extraction process. The effectiveness of heating is further heightened when a more polar solvent is employed. Thus, the following extraction solvents were used: 50% EtOH, 70% EtOH and 50% MeOH. Several researchers have stated that a binary solvent always improves the extraction efficiency compared to a monosolvent. Ethanol is the preferred choice due to its non-toxic nature, enabling the utilization of the extracted bioactive compounds in the food and pharmaceutical industries [39,40].

The plant material/solvent ratio is a very important parameter to consider in MAE extraction. Its optimal value is very specific to each extraction system and must therefore be determined experimentally. Thus, in order to assess the influence of the plant material/solvent ratio on the extraction of polyphenolic compounds, the plant material/solvent ratio was set at 1:20 (*m*/*v*) and 1:40 (*m*/*v*), respectively. Also, two sizes of plant material were used: coarse-sized plant material, denoted as G1, and very fine-sized plant material, denoted as G2. In total, 12 extracts were obtained for each LA, LL and G90 lavender species through MAE extraction. Each extraction was performed in triplicate (108 extracts).

The extract mass and the extraction yield for the LA, LL and G90 extracts obtained by the MAE extraction method are shown in Table 2. The highest extraction yield was obtained for the LA, LL and G90 extracts extracted with 70% EtOH. For the LA, LL and G90 extracts obtained with 50% MeOH, the extraction yield was higher when coarse-sized plant material (G1) was used, whereas in the case of the LA, LL and G90 extracts obtained with 50% EtOH and 70% EtOH, the extraction yield was higher when very small-sized plant material was used (G2). The study revealed a notably greater extraction of polyphenolic compounds using 50% MeOH in comparison to 50% EtOH. Similar to the patterns observed in the MAE and HD extraction of essential oils, the G90 lavender species exhibited the highest yield of polyphenolic compounds. An increase in the extraction yield when the solvent ratio was changed from 1:20 to 1:40 (*m*/*v*) was also noticed. Increasing the amount of the solvent increases the concentration difference between the plant material and the solvent, which acts as the driving force for the mass transfer. As a result of this, compounds will have a greater tendency to escape from the plant matrix (fed into the solvent by dissolution and diffusion) [41]. Therefore, the ratio 1:40 (*m*/*v*) was considered to be the optimal value for the plant material:solvent ratio. The extraction efficiency was predominantly influenced by changes in the composition of the ethanol–water binary solvent. The maximum extraction yield of the polyphenolic compounds was obtained at ethanol–water 70% (*v*/*v*), while the lowest yield was obtained at ethanol–water 50% (*v*/*v*). The reduced extraction yield observed at a higher proportion of water is probably due to non-hydro soluble compounds in the LA, LL, and G90 lavender species..

### 3.4. GC-MS/MS Analysis of Essential Oils

**Figure 2:**
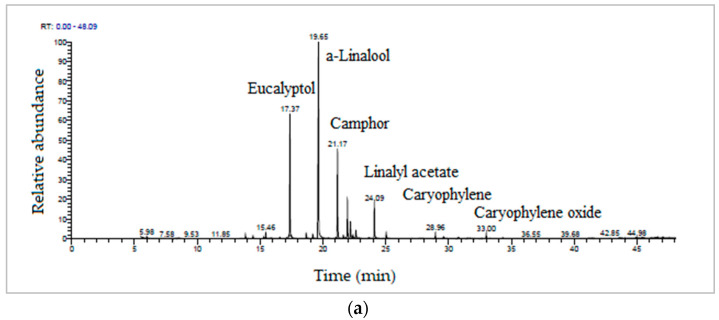

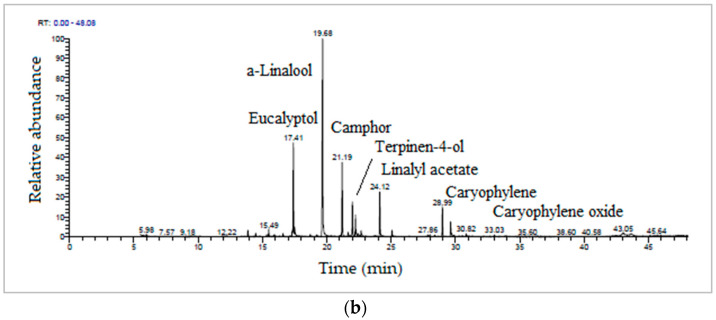
TIC chromatograms of (**a**) LA-MAE and (**b**) LA-HD essential oils.

The GC-MS/MS analysis was carried out for the essential oils extracted from the inflorescences of the LA, LL and G90 lavender species obtained by two different extraction methods: the MAE extraction method and the HD extraction method. Figure 2 shows the total ion abundance chromatogram (TIC) recorded by the GC-MS/MS technique of the LA essential oils obtained by the MAE extraction method and the HD extraction method, respectively, in which seven major bioactive compounds were identified by comparison with reference compounds from the MS spectral library of the GC-MS/MS software (Thermo Xcalibur 3.0). The most bioactive compounds identified, in order of retention time, were eucalyptol (17.37 min), α-linalool (19.65 min), camphor (21.17 min), terpinen-4-ol (22.23 min), linalyl acetate (24.09 min), caryophyllene (28.96 min) and caryophyllene oxide (33.00 min). The mean error of the retention time (RT) for the identified compounds was ±0.001–0.1 min.

**Figure 3:**
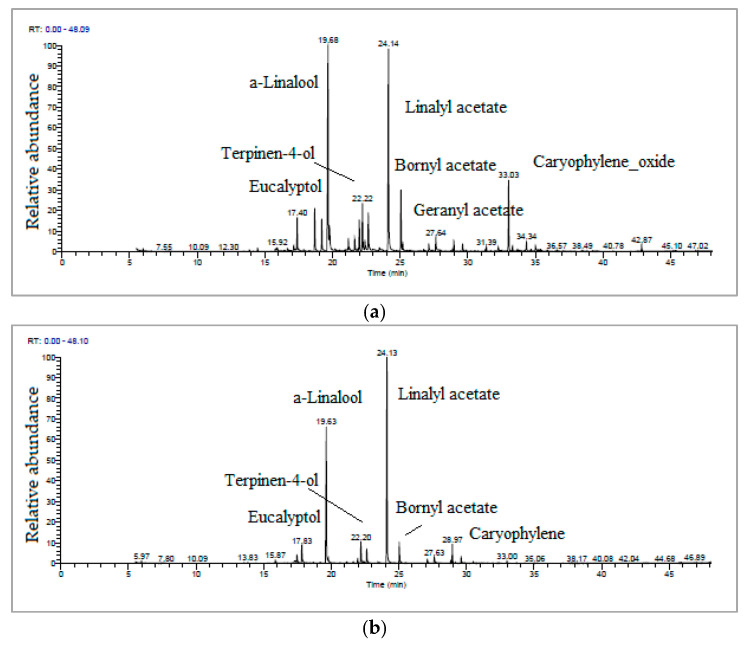
TIC chromatograms of (**a**) LL-MAE and (**b**) LL-HD essential oils.

The major bioactive compounds identified in LL-MAE essential oil, in order of retention time, were eucalyptol (17.40 min), α-linalool (19.68 min), terpinen-4-ol (22.22 min), linalyl acetate (24.14 min), bornyl acetate (25.21 min), geranyl acetate (27.64 min) and caryophyllene oxide (33.00 min). The major bioactive compounds identified in LL-HD essential oil, in order of retention time, were eucalyptol (17.83 min), α-linalool (19.63 min), terpinen-4-ol (22.20 min), linalyl acetate (24.13 min), bornyl acetate (25.21 min) and caryophyllene (28.97 min) (Figure 3). The caryophyllene sesquiterpenoid, known for its antioxidant, antiproliferative and antibacterial properties, was identified in higher percentages in the LL-HD essential oil than in the LL-MAE essential oil; instead, its derivative, caryophyllene oxide, which has the same beneficial properties for the human body, was identified in higher percentages in the LL-MAE essential oil than in the LL-HD essential oil (Figure 3b).

**Figure 4:**
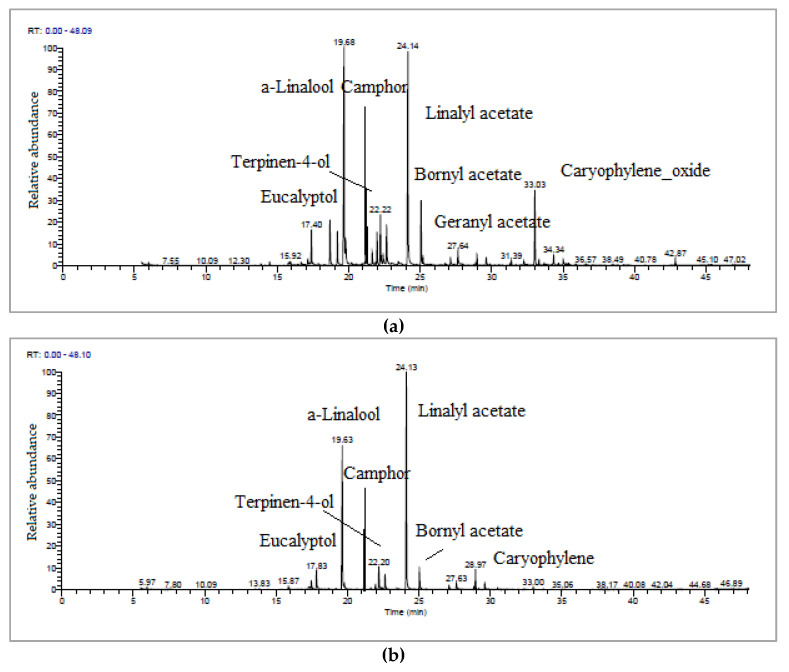
TIC chromatograms of (**a**) G90-MAE and (**b**) G90-HD essential oils.

The major bioactive compounds identified in the G90-MAE essential oil, according to the retention time, were eucalyptol (17.40 min), α-linalool (19.68 min), camphor (21.17 min), terpinene-4-ol (22.22 min), linalyl acetate (24.14 min), bornyl acetate (25.21 min), geranyl acetate (27.64 min) and caryophyllene oxide (33.00 min). The major bioactive compounds identified in G90-HD essential oil, in order of retention time, were eucalyptol (17.83 min), α-linalool (19.63 min), camphor (21.17 min), terpinene-4-ol (22.20 min), linalyl acetate (24.13 min), bornyl acetate (25.21 min) and caryophyllene (28.97 min) (Figure 4). The camphor terpenoid, which exhibits anti-inflammatory properties, was identified in G90 essential oils obtained by the two extraction methods: MAE extraction and HD extraction, similar to LA essential oils. Like LL essential oils, the caryophyllene sesquiterpenoid was identified in higher percentages in the G90-HD essential oil than in the G90-MAE essential oil, whereas its derivative, caryophyllene oxide, was identified in higher percentages in the essential oil G90-MAE than in the G90-HD essential oil.

The chemical composition of the LA, LL and G90 essential oils is shown in Table 3. In total, 41 bioactive compounds were identified, which were mainly hydrocarbons and hydrocarbon derivatives. In G90 essential oils, the major bioactive compounds (eucalyptol, α-linalool, camphor, terpinen-4-ol, linalyl acetate, bornyl acetate, geranyl acetate, caryophyllene and caryophyllene oxide) were identified with the highest abundance (%). Again, the MAE extraction method was found to be more effective than the HD extraction method, as the LA, LL and G90 essential oils were found to be richer in bioactive compounds in terms of concentration.

### 3.5. HPLC-DAD Analysis of Polyphenolic Compounds

For the identification and quantification of polyphenolic compounds from the LA, LL and G90 hydroalcoholic extracts, 21 reference compounds were used (tannic acid, gallic acid, protocatechuic acid, catechin, caffeic acid, chlorogenic acid, syringic acid, epicatechin, p-coumaric acid, ferulic acid, o-coumaric acid, ellagic acid, isoquercetin, rutin, rosmarinic acid, hyperoside, naringin, quercetin, luteolin, naringenin and kaempferol). To identify the polyphenolic compounds, the retention times of each LA, LL and G90 extract were compared with those of the reference compounds. The LA, LL and G90 hydroalcoholic extracts were analyzed at four different fixed λ wavelengths (255 nm, 280 nm, 325 nm and 355 nm), values that were chosen according to the λ_max_ value obtained when plotting molecular absorption spectra, from the λ range of 200–400 nm but also from data from the specialized literature [42].

The quantification of the polyphenolic compounds identified in the LA, LL and G90 hydroalcoholic extracts was carried out at the λ_max_ of each reference compound. Thus, a λ of 255 nm was used for protocatechuic acid and ellagic acid, a λ of 280 nm was used for tannic acid, gallic acid, catechin, syringic acid, epicatechin, o-coumaric acid, naringin and naringenin, a λ of 325 nm was used for caffeic acid, chlorogenic acid, p-coumaric acid, ferulic acid and rosmarinic acid and a λ of 355 nm was used for isoquercetin, rutin, hyperoside, quercetin, luteolin and kaempferol. Solutions of reference compounds with concentrations in the linear range 0.98–250 µg/mL were used for the calibration curves. The coefficients of determination (R^2^) of the linear regression functions for each compound have values larger than 0.996, which proves that the linear model is appropriate for our experimental data. The equations of the linear regression function for each reference compound were established by the correlation coefficient (r), which showed values around 0.999. For each reference compound, the LOD value was ≤0.98 μg/mL and the LOQ value was ≥0.98 μg/mL.

For the separation, identification and quantification of polyphenolic compounds from the LA, LL and G90 hydroalcoholic extracts, a concentration of 20 mg/mL was used. In total, 36 hydroalcoholic extracts of lavender were analyzed. All extracts were analyzed in triplicate (108 extracts), and the results were expressed as the mean ± standard error in Table 4, Table 5 and Table 6, respectively.

The data presented in Table 4 show the maximum concentration of each polyphenolic compound identified in the LA hydroalcoholic extracts, monitored in the UV range, at four different fixed λ wavelengths (255 nm, 280 nm, 325 nm and 355 nm). Thus, tannic acid, gallic acid, protocatechuic acid, catechin, caffeic acid, chlorogenic acid, syringic acid, p-coumaric acid, ferulic acid, o-coumaric acid, ellagic acid, isoquercetin, rutin, rosmarinic acid, hyperoside, naringin, quercetin, luteolin, naringenin and kaempferol were identified in all LA hydroalcoholic extracts. In total, 20 polyphenolic compounds were identified in the LA hydroalcoholic extracts. The flavonol kaempferol was identified only in the hydroalcoholic extracts, with 70% EtOH and with concentrations between 11,345.6 ± 62.0 and 20,582.8 ± 19.6 mg/kg. The major polyphenolic compounds identified in the LA hydroalcoholic extracts were: protocatechuic acid (3107.0 ± 17.0–11,025.0 ± 39.0 mg/kg), syringic acid (1017.3 ± 11.6–3847.3 ± 2.0 mg/kg), isoquercetin (972.6 ± 14.3–2776.2 ± 16.02 mg/kg), rutin (587.7 ± 6.7–1449.0 ± 61.4 mg/kg), hyperoside (1011.6 ± 76.0–5733.0 ± 166.9 mg/kg) and quercetin (1526.0 ± 51.0–9912.0 ± 123.0 mg/kg).

The data regarding the quantification of the polyphenolic compounds identified in the LL hydroalcoholic extracts are presented in Table 5. In total, 20 polyphenolic compounds were identified (tannic acid, gallic acid, protocatechuic acid, catechin, caffeic acid, chlorogenic acid, syringic acid, epicatechin, p-coumaric acid, ferulic acid, o-coumaric acid, ellagic acid, isoquercetin, rutin, rosmarinic acid, hyperoside, naringin, quercetin, luteolin and naringenin) in the LL hydroalcoholic extracts. Epicatechin was identified and quantified in the LL hydroalcoholic extracts (60.7 ± 4.3–395.1 ± 15.7 mg/kg) but not in the LA hydroalcoholic extracts; instead, kaempferol is not present in the LL hydroalcoholic extracts. The major polyphenolic compounds identified in the LL hydroalcoholic extracts were: ellagic acid (5642.8 ± 4.0 ± 19,572.4 ± 480.3 mg/kg), naringin (1606.0 ± 92.0–9162.3 ± 28.2 mg/kg) and rosmarinic acid (10,733.0 ± 33.0–39,841.0 ± 21.0 mg/kg).

The data presented in Table 6 show the maximum concentration of each polyphenolic compound identified in G90 hydroalcoholic extracts. Thus, tannic acid, gallic acid, protocatechuic acid, catechin, caffeic acid, chlorogenic acid, syringic acid, epicatechin, p-coumaric acid, ferulic acid, o-coumaric acid, ellagic acid, isoquercetin, rutin, rosmarinic acid, hyperoside, naringin, quercetin, luteolin and naringenin were identified in all G90 hydroalcoholic extracts. In total, 20 polyphenolic compounds have been identified in the G90 hydroalcoholic extracts. Flavonol kaempferol has not been identified in G90 hydroalcoholic extracts, similar to LL hydroalcoholic extracts; instead, epicatechin (209.9 ± 1.6–446.2 ± 11.0 mg/kg), a flavonoid that has anti-inflammatory and antitumor properties, is present. The majority of polyphenolic compounds identified in G90 hydroalcoholic extracts were: protocatechuic acid (6117.1 ± 33.7–130,65.3 ± 20.2 mg/kg), syringic acid (1118.0 ± 18.9–4074.8 ± 100.2 mg/kg), ellagic acid (6743.0 ± 30.0–22,528.1 ± 88.7 mg/kg), isoquercetin (1016.3 ± 6.2–2550.4 ± 32.1 mg/kg), rutin (477.9 ± 26.8–1748.9 ± 71.9 mg/kg), rosmarinic acid (17,432.1 ± 22.0–40,971.1 ± 114.4 mg/kg), hyperoside (1812.3 ± 46.2–6592.6 ± 45.9) and naringin (2009.1 ± 51.8–10,061.9 ± 108.2 mg/kg).

**Figure 5:**
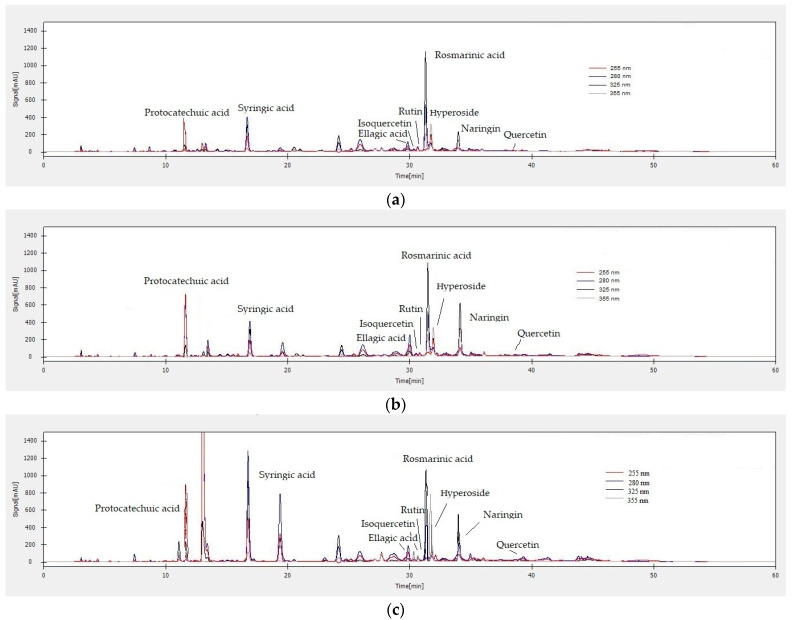
HPLC-DAD chromatograms of (**a**) LA, (**b**) LL and (**c**) G90 70% EtOH extracts, plant material particle size G2, plant material/solvent ratio 1:40, detected at a λ of 255 nm, 280 nm, 325 nm and 355 nm.

The chromatograms of the LA, LL and G90 hydroalcoholic extracts with λ detections of 255 nm, 280 nm, 325 nm and 355 nm are shown in Figure 5. Based on the retention time, 95% of the reference compounds in the LA, LL and G90 hydroalcoholic extracts were separated, identified and quantified. The most efficient extraction parameters used in the MAE extraction of bioactive compounds from the polyphenol class of the LA, LL and G90 lavender species were: 70% EtOH, plant material particle size G2 and solvent material ratio 1:40 > 50% MeOH, plant material particle size G1 and solvent material ratio 1:40 > 50% EtOH and particle size plant material G2 and solvent material ratio 1:40. The major polyphenolic compounds identified in the LA, LL and G90 hydroalcoholic extracts are: protocatechuic acid, syringic acid, ellagic acid, isoquercetin, rutin, rosmarinic acid, hyperoside and naringin (Figure 5). All reference compounds (tannic acid, gallic acid, protocatechuic acid, catechin, caffeic acid, chlorogenic acid, syringic acid, p-coumaric acid, ferulic acid, o-coumaric acid, ellagic acid, isoquercetin, rutin, rosmarinic acid, hyperoside, naringin, quercetin, luteolin, naringenin and kaempferol) used for the HPLC-DAD analysis of LA, LL and G90 lavender extracts were found in the greatest quantity in the G90 hydroalcoholic extracts. In contrast, hydroxybenzoic acids (tannic acid, gallic acid and protocatechuic acid) and hydroxybenzoic acid derivatives (syringic acid) have been identified in higher quantities in LA hydroalcoholic extracts. At the same time, hydroxycinnamic acids (caffeic acid, p-coumaric acid, o-coumaric acid and ferulic acid), but also derivatives of hydroxycinnamic acids (chlorogenic acid and rosmarinic acid), were identified in higher quantities in LL hydroalcoholic extracts. Ellagic acid and the flavonoids naringin and naringenin were identified in higher quantities in LL hydroalcoholic extracts, and the flavonoids isoquercetin, rutin, hyperoside, quercetin and luteolin were found in higher amounts in LA hydroalcoholic extracts.

### 3.6. Antioxidant Potential of Bioactive Compounds

#### 3.6.1. Antioxidant Potential by DPPH Free Radical Inhibition

**Figure 6:**
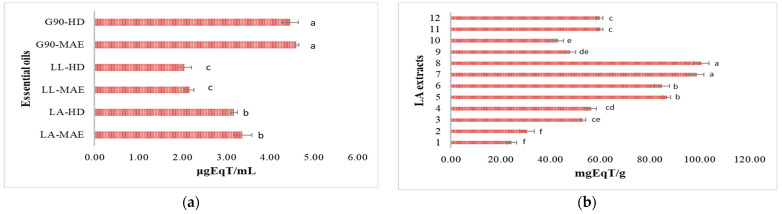

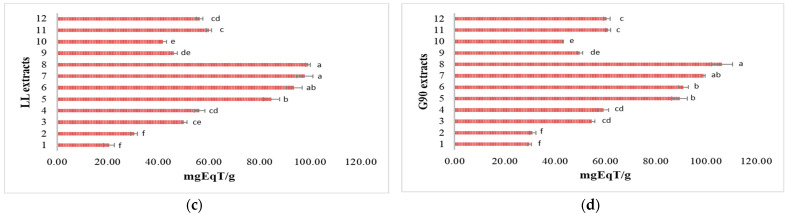
DPPH antioxidant potential expressed as antioxidant activity equivalents of (**a**) LA, LL and G90 essential oils and (**b**) LA, (**c**) LL and (**d**) G90 hydroalcoholic extracts. LA-MAE—*L. angustifolia* MAE essential oil, LA-HD—*L. angustifolia* HD essential oil, LL-MAE—*L. latifolia* MAE essential oil, LL-HD—*L. latifolia* HD essential oil, G90-MAE—*George 90* MAE essential oil and G90-HD—*George 90* HD essential oil. 1—EtOH 50%, plant material/solvent ratio at 1:20 (*m*/*v*), G1 coarse-sized plant material; 2—EtOH 50%, plant material/solvent ratio at 1:20 (*m*/*v*), G2 fine-sized plant material; 3—EtOH 50%, plant material/solvent ratio at 1:40 (*m*/*v*), G1 coarse-sized plant material; 4—EtOH 50%, plant material/solvent ratio at 1:40 (*m*/*v*), G2 fine-sized plant material; 5—EtOH 70%, plant material/solvent ratio at 1:20 (*m*/*v*), G1 coarse-sized plant material; 6—EtOH 70%, plant material/solvent ratio at 1:20 (*m*/*v*), G2 fine-sized plant material; 7—EtOH 70%, plant material/solvent ratio at 1:40 (*m*/*v*), G1 coarse-sized plant material; 8—EtOH 70%, plant material/solvent ratio at 1:40 (*m*/*v*), G2 fine-sized plant material; 9—MeOH 50%, plant material/solvent ratio at 1:20 (*m*/*v*), G1 coarse-sized plant material; 10—MeOH 50%, plant material/solvent ratio at 1:20 (*m*/*v*), G2 fine-sized plant material; 11—MeOH 50%, plant material/solvent ratio at 1:40 (*m*/*v*), G1 coarse-sized plant material; 12—MeOH 50%, plant material/solvent ratio at 1:40 (*m*/*v*), G2 fine-sized plant material. Values are means ± SD, *n* = 3 per treatment group. Means in a bar without a common superscript letter differ (*p* < 0.05), as analyzed by one-way ANOVA and the TUKEY test.

The antioxidant potential of lavender samples (essential oils and hydroalcoholic extracts) was expressed as a percentage of DPPH free radical inhibition and Trolox equivalents. The DPPH stable free radical reduction to a yellow color from a purple color [43] was obtained for all the LA, LL and G90 lavender samples, representing a decrease in absorbance at a λ of 517 nm. The results are reported in Figure S1 (Supplementary Materials) and Figure 6. The antioxidant potential of LA, LL and G90 crude essential oils expressed as a percentage of DPPH free radical inhibition are shown in Figure S1a, and the antioxidant activity equivalents are shown in Figure 6a. The highest percentage of DPPH free radical inhibition is represented by G90-MAE and G90-HD essential oils, in descending order: 94.65 ± 1.75% and 89.22 ± 2.01%, followed by LA-MAE (75.18 ± 1.05%) and LA-HD (68.14 ± 0.95%), LL-MAE (72.03 ± 2.10%) and LL-HD (66.56 ± 0.65%), respectively (Figure S1a). The values expressed as Trolox equivalents of LA, LL and G90 essential oils were as follows: G90-MAE (4.60 ± 0.05 μgEqT/mL) > G90-HD (4.46 ± 0.18 μgEqT/mL) > LA-MAE (3.37 ± 0.21 μgEqT/mL) > LA-HD (3.18 ± 0.08 μgEqT/mL) > LL-MAE (2.16 ± 0.11 μgEqT/mL) > LL-HD (2.05 ± 0.16 μgEqT/mL) (Figure 6a).

Figure S1b shows the antioxidant potential of LA hydroalcoholic extracts obtained by MAE extraction, expressed as a percentage of DPPH free radical inhibition. The highest percentage of DPPH free radical inhibition is represented by LA 70% EtOH extracts with a 1:40 ratio (*m*/*v*): 98.24 ± 1.23% (G2) > 94.12 ± 1.99% (G1). The lowest percentage of DPPH free radical inhibition is represented by LA 50% EtOH extracts with a 1:40 ratio (*m*/*v*): 68.85 ± 0.59% (G2) > 66.07 ± 1.17% (G1). The LA 50% MeOH extracts with a 1:40 ratio (*m*/*v*) present the following percentages of DPPH free radical inhibition: 75.36 ± 0.79% (G1) > 69.26 ± 0.98% (G2). The values expressed as Trolox equivalents are: LA 70% EtOH extracts with a 1:40 ratio (*m*/*v*): 100.71 ± 3.03 mgEqT/g (G2) > 98.90 ± 2.73 mgEqT/g (G1), followed by LA 50% MeOH extracts with a 1:40 ratio (*m*/*v*): 60.12 ± 0.97 mgEqT/g (G1) > 59.84 ± 1.31 mgEqT/g (G2) and followed by LA 50% EtOH extracts with a 1:40 ratio (*m*/*v*): 56.45 ± 2.11 mgEqT/g (G2) > 53.06 ± 1.09 mgEqT/g (G1) (Figure 6b).

The antioxidant potential of LL hydroalcoholic extracts obtained by MAE extraction, expressed as a percentage of DPPH free radical inhibition, is shown in Figure S1c. The highest percentage of DPPH free radical inhibition is represented by LL 70% EtOH extracts with a 1:40 ratio (*m*/*v*): 97.15 ± 2.11% (G2) > 93.37 ± 0.89% (G1). The lowest percentage of DPPH free radical inhibition is represented by LL 50% EtOH extracts with a 1:40 ratio (*m*/*v*): 67.08 ± 0.86% (G2) > 65.75 ± 2.01% (G1). The LL 50% MeOH extracts with a 1:40 ratio (*m*/*v*) present the following percentages of DPPH free radical inhibition: 73.16 ± 1.88% (G1) > 67.32 ± 1.12% (G2). The values expressed as Trolox equivalents are: LL 70% EtOH extracts with a 1:40 ratio (*m*/*v*): 99.14 ± 0.98 mgEqT/g (G2) > 97.87 ± 3.23 mgEqT/g (G1), followed by LL 50% MeOH extracts with a 1:40 ratio (*m*/*v*): 59.94 ± 0.97 mgEqT/g (G1) > 56.35 ± 1.31 mgEqT/g (G2) and followed by LL 50% EtOH extracts with a 1:40 ratio (*m*/*v*): 56.20 ± 2.11 mgEqT/g (G2) > 50.13 ± 1.09 mgEqT/g (G1) (Figure 6c).

Figure S1d shows the antioxidant potential of G90 hydroalcoholic extracts obtained by MAE extraction, expressed as a percentage of DPPH free radical inhibition. Like the LA and LL hydroalcoholic extracts, the highest percentage of DPPH free radical inhibition is represented by G90 70% EtOH extracts with a ratio of 1:40 (*m*/*v*): 99.44 ± 3.05% (G2) > 95.63 ± 1.75% (G1). The lowest percentage of DPPH free radical inhibition is represented by G90 50% EtOH extracts with a 1:40 ratio (*m*/*v*): 75.27 ± 1.25% (G2) > 72.46 ± 1.31% (G1). The G90 50% MeOH extracts with a 1:40 ratio (*m*/*v*) present the following percentages of DPPH free radical inhibition: 76.36 ±2.04% (G1) > 75.80 ± 1.60% (G2). The values expressed as Trolox equivalents are: G90 70% EtOH extracts with a 1:40 ratio (*m*/*v*): 106.30 ± 4.24 mgEqT/g (G2) > 99.07 ± 0.74 mgEqT/g (G1), followed by G90 50% MeOH extracts with a 1:40 ratio (*m*/*v*): 61.09 ± 0.97 mgEqT/g (G1) > 60.49 ± 1.31 mgEqT/g (G2) and followed by G90 50% EtOH extracts with a 1:40 ratio (*m*/*v*): 59.22 ± 2.11 mgEqT/g (G2) > 54.67 ± 1.09 mgEqT/g (G1) (Figure 6d).

#### 3.6.2. Antioxidant Potential by Inhibiting the ABTS^●+^ Cationic Radical

**Figure 7:**
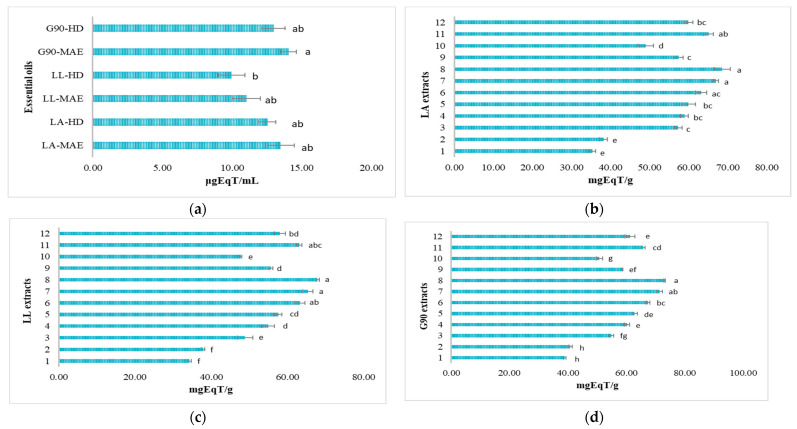
ABTS antioxidant potential expressed as the antioxidant activity equivalents of (**a**) LA, LL and G90 essential oils and (**b**) LA, (**c**) LL and (**d**) G90 hydroalcoholic extracts. LA-MAE—*L. angustifolia* MAE essential oil, LA-HD—*L. angustifolia* HD essential oil, LL-MAE—*L. latifolia* MAE essential oil, LL-HD—*L. latifolia* HD essential oil, G90-MAE—*George 90* MAE essential oil and G90-HD—*George 90* HD essential oil. 1—EtOH 50%, plant material/solvent ratio at 1:20 (*m*/*v*), G1 coarse-sized plant material; 2—EtOH 50%, plant material/solvent ratio at 1:20 (*m*/*v*), G2 fine-sized plant material; 3—EtOH 50%, plant material/solvent ratio at 1:40 (*m*/*v*), G1 coarse-sized plant material; 4—EtOH 50%, plant material/solvent ratio at 1:40 (*m*/*v*), G2 fine-sized plant material; 5—EtOH 70%, plant material/solvent ratio at 1:20 (*m*/*v*), G1 coarse-sized plant material; 6—EtOH 70%, plant material/solvent ratio at 1:20 (*m*/*v*), G2 fine-sized plant material; 7—EtOH 70%, plant material/solvent ratio at 1:40 (*m*/*v*), G1 coarse-sized plant material; 8—EtOH 70%, plant material/solvent ratio at 1:40 (*m*/*v*), G2 fine-sized plant material; 9—MeOH 50%, plant material/solvent ratio at 1:20 (*m*/*v*), G1 coarse-sized plant material; 10—MeOH 50%, plant material/solvent ratio at 1:20 (*m*/*v*), G2 fine-sized plant material; 11—MeOH 50%, plant material/solvent ratio at 1:40 (*m*/*v*), G1 coarse-sized plant material; 12—MeOH 50%, plant material/solvent ratio at 1:40 (*m*/*v*), G2 fine-sized plant material. Values are presented as means ± SD, *n* = 3 per treatment group. Data without a common superscript letter differ (*p* < 0.05), as analyzed by one-way ANOVA and the TUKEY test.

The antioxidant potential of lavender samples (essential oils and hydroalcoholic extracts) was expressed as a percentage of ABTS^●+^ cationic radical inhibition and Trolox equivalents. The ABTS^●+^ cationic radical exhibits a change in color from intensely turquoise to slightly yellow, with an absorbance at λ of 734 nm [44]. The results are reported in Figure S2 (Supplementary Materials) and Figure 7. The antioxidant potential of LA, LL and G90 crude essential oils expressed as a percentage of ABTS^●+^ cationic radical inhibition are shown in Figure S2a, and the antioxidant activity equivalents are shown in Figure 7a. The highest percentage of ABTS^●+^ cationic radical inhibition is represented by G90-MAE (99.49 ± 2.15%) and G90-HD (96.22 ± 0.97%) essential oils, like the antioxidant potential of LA, LL and G90 essential oils by DPPH free radical inhibition, followed by LA-MAE (91.35 ± 1.09%) and LA-HD (87.67 ± 1.73%), LL-MAE (88.42 ± 1.61%) and LL-HD (82.69 ± 1.24%), respectively (Figure S2a). The values expressed as Trolox equivalents of LA, LL and G90 essential oils were as follows: G90-MAE (14.07 ± 0.56 μgEqT/mL) > G90-HD (12.97 ± 0.82 μgEqT/mL) > LA-MAE (13.48 ± 0.99 μgEqT/mL) > LA-HD (12.53 ± 0.62 μgEqT/mL) > LL-MAE (11.03 ± 1.01 μgEqT/mL) > LL-HD (9.98 ± 0.96 μgEqT/mL) (Figure 7a).

Figure S2b shows the antioxidant potential of LA hydroalcoholic extracts obtained by MAE extraction, expressed as a percentage of ABTS^●+^ cationic radical inhibition. The highest percentage of ABTS^●+^ cationic radical inhibition is represented by LA 70% EtOH extracts with a 1:40 ratio (*m*/*v*): 95.98 ± 2.04% (G2) > 87.06 ± 0.79% (G1). The lowest percentage of ABTS^●+^ cationic radical inhibition is represented by LA 50% EtOH extracts with a 1:40 ratio (*m*/*v*): 63.94 ± 1.43% (G2) > 57.62 ± 1.62% (G1). The LA 50% MeOH extracts with a 1:40 ratio (*m*/*v*) present the following percentages of ABTS^●+^ cationic radical inhibition: 68.63 ± 0.77% (G1) > 67.32 ± 1.05% (G2). The values expressed as Trolox equivalents are: LA 70% EtOH extracts with a 1:40 ratio (*m*/*v*): 68.55 ± 2.00 mgEqT/g (G2) > 66.89 ± 0.73 mgEqT/g (G1), followed by LA 50% MeOH extracts with a 1:40 ratio (*m*/*v*): 65.05 ± 1.23 mgEqT/g (G1) > 59.90 ± 1.11 mgEqT/g (G2) and followed by LA 50% EtOH extracts with a 1:40 ratio (*m*/*v*): 58.90 ± 1.00 mgEqT/g (G2) > 57.26 ± 0.99 mgEqT/g (G1) (Figure 7b).

The antioxidant potential of LL hydroalcoholic extracts obtained by MAE extraction, expressed as a percentage of ABTS^●+^ cationic radical inhibition, is shown in Figure S2c. The highest percentage of ABTS^●+^ cationic radical inhibition is represented by LL 70% EtOH extracts with a 1:40 ratio (*m*/*v*): 93.89 ± 2.01% (G2) > 83.57 ± 0.97% (G1). The lowest percentage of ABTS^●+^ cationic radical inhibition is represented by LL 50% EtOH extracts with a 1:40 ratio (*m*/*v*): 59.98 ± 0.86% (G2) > 50.54 ± 2.01% (G1). The LL 50% MeOH extracts with a 1:40 ratio (*m*/*v*) present the following percentages of ABTS^●+^ cationic radical inhibition: 68.07 ± 1.88% (G1) > 61.47 ± 1.12% (G2). The values expressed as Trolox equivalents are: LL 70% EtOH extracts with a 1:40 ratio (*m*/*v*): 67.87 ± 0.43 mgEqT/g (G2) > 65.31 ± 1.30 mgEqT/g (G1), followed by LL 50% MeOH extracts with a 1:40 ratio (*m*/*v*): 63.03 ± 0.77 mgEqT/g (G1) > 57.95 ± 1.45 mgEqT/g (G2) and followed by LL 50% EtOH extracts with a 1:40 ratio (*m*/*v*): 54.91 ± 1.67 mgEqT/g (G2) > 48.82 ± 2.00 mgEqT/g (G1) (Figure 7c).

Figure S2d shows the antioxidant potential of G90 hydroalcoholic extracts obtained by MAE extraction, expressed as a percentage of ABTS^●+^ cationic radical inhibition. Like the LA and LL hydroalcoholic extracts, the highest percentage of ABTS^●+^ cationic radical inhibition is represented by G90 70% EtOH extracts with a ratio of 1:40 (*m*/*v*): 99.64 ± 1.05% (G2) > 94.01 ± 0.87% (G1). The lowest percentage of ABTS^●+^ cationic radical inhibition is represented by G90 50% EtOH extracts with a 1:40 ratio (*m*/*v*): 69.77 ± 1.33% (G2) > 66.91 ± 1.41% (G1). The G90 50% MeOH extracts with a 1:40 ratio (*m*/*v*) present the following percentages of ABTS^●+^ cationic radical inhibition: 77.06 ± 1.72% (G1) > 70.54 ± 1.05% (G2). The values expressed as Trolox equivalents are: G90 70% EtOH extracts with a 1:40 ratio (*m*/*v*): 73.12 ± 0.29 mgEqT/g (G2) > 71.36 ± 1.04 mgEqT/g (G1), followed by G90 50% MeOH extracts with a 1:40 ratio (*m*/*v*): 65.82 ± 0.66 mgEqT/g (G1) > 61.23 ± 1.75 mgEqT/g (G2) and followed by G90 50% EtOH extracts with a 1:40 ratio (*m*/*v*): 60.25 ± 0.91 mgEqT/g (G2) > 54.87 ± 0.89 mgEqT/g (G1) (Figure 7d).

#### 3.6.3. Antioxidant Potential through Ferric Ion Reduction (FRAP)

**Figure 8:**
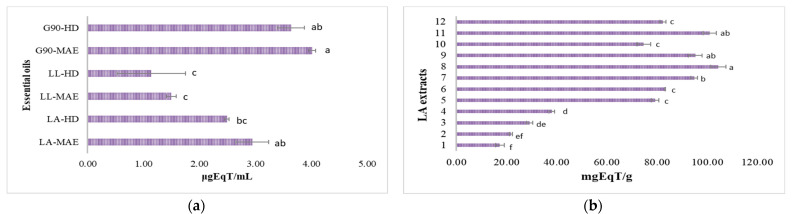

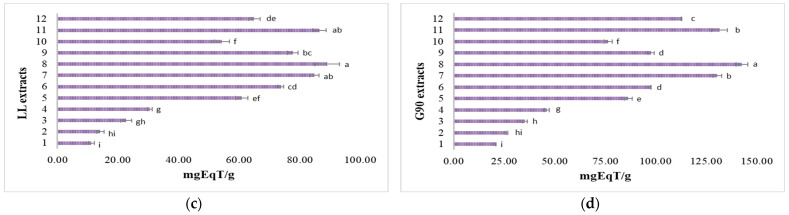
FRAP antioxidant potential expressed as antioxidant activity equivalents of (**a**) LA, LL and G90 essential oils and (**b**) LA, (**c**) LL and (**d**) G90 hydroalcoholic extracts. LA-MAE—*L. angustifolia* MAE essential oil, LA-HD—*L. angustifolia* HD essential oil, LL-MAE—*L. latifolia* MAE essential oil, LL-HD—*L. latifolia* HD essential oil, G90-MAE—*George 90* MAE essential oil and G90-HD—*George 90* HD essential oil. 1—EtOH 50%, plant material/solvent ratio at 1:20 (*m*/*v*), G1 coarse-sized plant material; 2—EtOH 50%, plant material/solvent ratio at 1:20 (*m*/*v*), G2 fine-sized plant material; 3—EtOH 50%, plant material/solvent ratio at 1:40 (*m*/*v*), G1 coarse-sized plant material; 4—EtOH 50%, plant material/solvent ratio at 1:40 (*m*/*v*), G2 fine-sized plant material; 5—EtOH 70%, plant material/solvent ratio at 1:20 (*m*/*v*), G1 coarse-sized plant material; 6—EtOH 70%, plant material/solvent ratio at 1:20 (*m*/*v*), G2 fine-sized plant material; 7—EtOH 70%, plant material/solvent ratio at 1:40 (*m*/*v*), G1 coarse-sized plant material; 8—EtOH 70%, plant material/solvent ratio at 1:40 (*m*/*v*), G2 fine-sized plant material; 9—MeOH 50%, plant material/solvent ratio at 1:20 (*m*/*v*), G1 coarse-sized plant material; 10—MeOH 50%, plant material/solvent ratio at 1:20 (*m*/*v*), G2 fine-sized plant material; 11—MeOH 50%, plant material/solvent ratio at 1:40 (*m*/*v*), G1 coarse-sized plant material; 12—MeOH 50%, plant material/solvent ratio at 1:40 (*m*/*v*), G2 fine-sized plant material. Values are means ± SD, *n* = 3 per treatment group. Means in a bar without a common superscript letter differ (*p* < 0.05), as analyzed by one-way ANOVA and the TUKEY test.

The decrease in absorbance is proportional to the antioxidant content of the lavender samples (essential oils and hydroalcoholic extracts), based on the ability of different antioxidant compounds to reduce Fe^3+^ to a more stable form, Fe^2+^, in the presence of TPTZ, forming an intense blue color complex, Fe^2+^-TPTZ [45]. The results are reported in Figure 8. The antioxidant potential of LA, LL and G90 crude essential oils through the ferric ion reduction (FRAP) assay, expressed as Trolox equivalents, is shown in Figure 8a. The values expressed as Trolox equivalents of LA, LL and G90 essential oils were as follows: G90-MAE (4.01 ± 0.06 μgEqT/mL) > G90-HD (3.64 ± 0.24 μgEqT/mL) > LA-MAE (2.95 ± 0.29 μgEqT/mL) > LA-HD (2.49 ± 0.04 μgEqT/mL) > LL-MAE (1.50 ± 0.09 μgEqT/mL) > LL-HD (1.14 ± 0.61 μgEqT/mL).

The antioxidant potential of LA, LL and G90 hydroalcoholic extracts through the ferric ion reduction (FRAP) assay, expressed as Trolox equivalents, is shown in Figure 8b–d. The values expressed as Trolox equivalents of LA, LL and G90 hydroalcoholic extracts were, in descending order, as follows: G90 hydroalcoholic extracts (20.82 ± 0.16–142.66 ± 2.96 mgEqT/g, Figure 8d) > LA hydroalcoholic extracts (17.38 ± 1.73–104.48 ± 3.02 mgEqT/g, Figure 8b) > LL hydroalcoholic extracts (11.28 ± 0.95–89.12 ± 4.04 mgEqT/g, Figure 8c). Regarding the solvent extractions, as in the case of the DPPH and ABTS assays, 70% EtOH lavender extracts with a 1:40 ratio (*m*/*v*) showed a higher antioxidant potential, followed by 50% MeOH lavender extracts with a 1:40 ratio (*m*/*v*) and 50% EtOH lavender extracts with a 1:40 ratio (*m*/*v*). Thus, the antioxidant potential of LA hydroalcoholic extracts through the FRAP assay, expressed as Trolox equivalents, was as follows: 104.48 ± 3.02 mgEqT/g (70% EtOH, with a 1:40 ratio, G2) > 100.97± 2.57 mgEqT/g (50% MeOH, with a 1:40 ratio, G1) > 38.11 ± 1.09 mgEqT/g (50% EtOH, with a 1:40 ratio, G2). The antioxidant potential of LL hydroalcoholic extracts through the FRAP assay, expressed as Trolox equivalents, was as follows: 89.12 ± 4.04 mgEqT/g (70% EtOH, with a 1:40 ratio, G2) > 86.55 ± 2.18 mgEqT/g (50% MeOH, with a 1:40 ratio, G1) > 30.49 ± 0.78 mgEqT/g (50% EtOH, with a 1:40 ratio, G2). The antioxidant potential of G90 hydroalcoholic extracts through the FRAP assay, expressed as Trolox equivalents, was as follows: 142.66 ± 2.96 mgEqT/g (70% EtOH, with a 1:40 ratio, G2) > 131.59 ± 3.93 mgEqT/g (50% MeOH, with a 1:40 ratio, G1) > 45.95 ± 1.23 mgEqT/g (50% EtOH, with a 1:40 ratio, G2).

### 3.7. Antimicrobial Potential of Bioactive Compounds against Gram-Positive Bacteria

This study aimed to evaluate the antimicrobial potential of the LA, LL and G90 crude essential oils. The antibacterial potential of the LA, LL and G90 crude essential oils and ciprofloxacin has been evaluated by determining their MIC and MBC values by using the broth microdilution method. As shown in Table 7, the investigated LA, LL and G90 crude essential oils exhibited notable antibacterial activity against *Gram-positive* bacteria: the *B. cereus* and *B. subtilis* strains tested, with MIC values ranging from 2.5 to 10%. Ciprofloxacin, the standard drug tested, exhibited antibacterial activity against the *B. cereus* and *B. subtilis* strains, with MIC and MBC values of 0.3 µg/mL and 0.2 µg/mL, respectively. All LA, LL and G90 crude essential oils showed significant antibacterial activity against *Gram-positive* bacteria species. The MBC values of the LA, LL and G90 crude essential oils were similar or even higher than the corresponding MIC values, with an MIC/MBC ratio very close to 1, confirming their bactericidal activity [46]. The best antibacterial activity (MIC 2.5%) was shown against *B. subtilis*. The weaker activity was observed against *B. cereus* (MIC 5%) (Table 7). The LA, LL and G90 crude essential oils can thus be used as effective antibacterial agents against several *Gram-positive* bacterial species. The strong antibacterial activity of the LA, LL and G90 crude essential oils can be attributed to a high concentration of their oxygenated terpenes (eucalyptol, α-linalool, terpinen-4-ol and linalyl acetate) [47]. The results indicated that essential oils have antimicrobial activity in their airborne evaporative state [48].

### 3.8. Antimicrobial Potential of Bioactive Compounds against Different Type of Fungus

The antifungal potential has been evaluated for the LA, LL and G90 crude essential oils and fluconazole by determining their MIC and MFC values by using the broth microdilution method. The LA, LL and G90 crude essential oils showed a wide-spectrum antifungal activity. The MIC and MFC values of LA, LL and G90 crude essential oils are shown in Table 8. The LA, LL and G90 crude essential oils were very active against *A. brasiliensis*, *F. oxysporum* and *P. expansum*, with MIC values of 1.25% for *A. brasiliensis*, 2.5% for *F. oxysporum* and 40% for *P. expansum*. The antifungal activity of the LA, LL and G90 crude essential oils resulted from a synergistic effect of the major compounds [47]. For the fungal strain tested (*A. brasiliensis*, *F. oxysporum* and *P. expansum*), the MIC was equivalent to the MFC, suggesting a fungicidal potential of the LA, LL and G90 crude essential oils. Fluconazole, the standard drug tested, exhibited antifungal activity against *F. oxysporum*, with MIC and MFC values of 100.0 µg/mL.

### 3.9. Antiproliferative Potential of Bioactive Compounds

**Figure 9:**
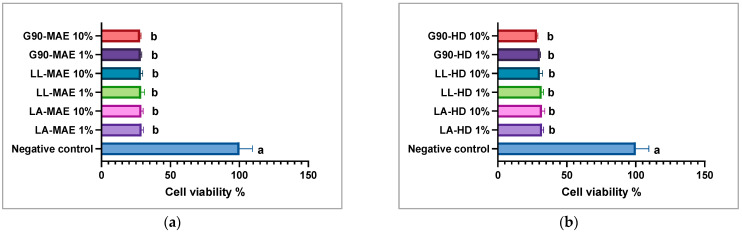
Antiproliferative potential of LA, LL and G90 essential oils against the HeLa tumoral cell line (**a**,**b**). Data are the mean ± SD of four replicates per condition. LA-MAE—*L. angustifolia* MAE essential oil, LA-HD—*L. angustifolia* HD essential oil, LL-MAE—*L. latifolia* MAE essential oil, LL-HD—*L. latifolia* HD essential oil, G90-MAE—*George 90* MAE essential oil and G90-HD—*George 90* HD essential oil. Values are means ± SD, *n* = 3 per treatment group. Means in a bar without a common superscript letter differ (*p* < 0.05), as analyzed by one-way ANOVA and the TUKEY test.

The essential oils contain valuable secondary metabolites that can be used in the treatment of colon, breast, liver and prostate tumors without severely modifying the normal cells [49]. An MTT assay showed the antiproliferative potential of LA, LL and G90 essential oils in the HeLa tumoral cell line. Different quantities (1 and 10%) of LA, LL and G90 essential oils were applied to the HeLa tumoral cell line for 24 h. According to data obtained by the MTT assay, a different dose of LA, LL and G90 essential oils yielded the same cell viability results in the HeLa tumoral cell line (Figure 9). Moreover, these results indicated that LA, LL and G90 essential oils can effectively reduce cells proliferation at lower doses. This property of the LA, LL and G90 essential oils may be due to their secondary metabolite composition. Principal components, eucalyptol, α-linalool, terpinen-4-ol and linalyl acetate, have been shown to demonstrate antitumoral potential on various cell lines in other studies [50,51]. According to some studies, LA, LL and G90 essential oils have a cytotoxic effect that is associated with both necrosis and apoptosis [23]. The cell morphological changes induced by the LA, LL and G90 essential oils are shown in Figure S3 (Supplementary Materials).

## 4. Discussion

Bioactive compounds could be considered as a very promising beginning for the development of new and novel therapeutic agents [52]. This study investigated the chemical composition and biological properties of LA, LL and G90 lavender species. MAE extraction proved to be the most efficient extraction method for the extraction of essential oils and polyphenolic compounds from the LA, LL and G90 lavender species. Different parameters of the MAE extraction process, namely, the size of the plant material (G1 and G2), the plant material/solvent ratio (1:20 and 1:40), different solvents (EtOH and MeOH) and different solvent proportions (50% and 70%), have been successfully used for the extraction of polyphenolic compounds. The highest extraction yield was obtained for LA, LL and G90 hydroalcoholic extracts extracted with 70% EtOH. It was found that a much higher quantity of polyphenolic compounds was extracted with 50% MeOH compared to 50% EtOH, and the 1:40 (*m*/*v*) ratio was the optimal value for the plant material/solvent ratio. In the case of both the MAE extraction and HD extraction of LA, LL and G90 essential oils, as well as in the case of the MAE extraction of polyphenolic compounds, the highest extraction yield was obtained for the G90 lavender species George 90 (G90 > LL > LA). The LA, LL and G90 lavender species can be a considerable source of natural compounds. This study demonstrates that the MAE extraction method can be used to obtain compounds that are present in small quantities and to prevent their possible degradation when conventional extraction methods are used.

In total, 41 bioactive compounds were identified in essential oils extracted from the inflorescences of the LA, LL and G90 lavender species, which were mainly hydrocarbons and hydrocarbon derivatives. G90 essential oils identified the majority bioactive compounds (eucalyptol, α-linalool, camphor, terpinen-4-ol, linalyl acetate, bornyl acetate, geranyl acetate, caryophyllene and caryophyllene oxide) with the highest abundance (%), compared to LA essential oils (eucalyptol, α-linalool, camphor, terpinen-4-ol, linalyl acetate, caryophyllene and caryophyllene oxide) and LL (eucalyptol, α-linalool, terpinen-4-ol, linalyl acetate, bornyl acetate, geranyl acetate, caryophyllene and caryophyllene oxide). The MAE extraction method proved to be more efficient than the HD extraction method because LA, LL and G90 essential oils were found to be richer in bioactive compounds in terms of concentration. Several research studies on the phytochemical composition of different extracts (alcoholic, hydroalcoholic), obtained by different extraction methods (Soxhelt extraction, ultrasound-assisted extraction, microwave assisted extraction) and using as plant material different lavender species (L. *angustifolia, L. latifolia*, *L. x intermedia*), have reported the presence of phenolic acids such as protocatechuic acid, gallic acid, rosmarinic acid, caffeic acid, p-coumaric acid and ferulic acid and flavonoids such as apigenin, luteolin, catechin, epicatechin, naringenin and myricetin. The concentration of each of these polyphenolic compounds varies according to different species and depends on the genotype, geographical origin, climatic conditions, growing conditions, harvesting time and extraction method [53,54,55]. The LA, LL and G90 hydroalcoholic extracts are a rich source of bioactive compounds belonging to the polyphenol class. For the HPLC-DAD analysis of polyphenolic compounds present in LA, LL and G90 hydroalcoholic extracts, 21 reference compounds were used (tannic acid, gallic acid, protocatechuic acid, catechin, caffeic acid, chlorogenic acid, syringic acid, epicatechin, p-coumaric acid, ferulic acid, o-coumaric acid, ellagic acid, isoquercetin, rutin, rosmarinic acid, hyperoside, naringin, quercetin, luteolin, naringenin and kaempferol), and 20 of these (95%) were identified and quantified in all of the LA, LL and G90 hydroalcoholic extracts. Flavonol kaempferol has only been identified in LA hydroalcoholic extracts (70% EtOH), with concentrations ranging from 11,345.6 ± 62.0 mg/kg to 20,582.8 ± 19.6 mg/kg, whereas kaempferol is not present in the LL and G90 hydroalcoholic extracts. Epicatechin has been identified and quantified in LL hydroalcoholic extracts (60.7 ± 4.3–395.1 ± 15 mg/kg) and G90 (209.9 ± 1.6–446.2 ± 11.0 mg/kg) but not in LA hydroalcoholic extracts.

For every plant species, there is a different optimal stage of harvesting to have the maximum quantity of bioactive compounds [21]. Still, the LA, LL and G90 lavender species’ harvest time must generally be in the afternoon, after the plants accumulate bioactive compounds with antioxidant activity because of the power sun lights during the day. The highest antioxidant potential of both LA, LL and G90 crude essential oils and hydroalcoholic extracts was, in descending order, as follows: G90 > LA > LL lavender species. It is known that lavender essential oils and hydroalcoholic extracts have antioxidant potential, as they scavenge DPPH radical molecules. Still, their capacity is relatively weaker than that of vitamin C and Trolox used as antioxidant reference compounds [56]. Generally, the antioxidant activity of the LA, LL and G90 crude essential oils is related to the major active compounds in the essential oil, such as linalool and its derivatives [57]. The antioxidant activity of the LA, LL and G90 hydroalcoholic extracts is related to the major polyphenolic compounds, such as protocatechuic acid, syringic acid, ellagic acid, isoquercetin, rutin, rosmarinic acid, hyperoside and naringin [58]. Furthermore, there was a significant difference in all LA, LL and G90 lavender samples for the antioxidant potential analyzed with all the antioxidant methods (DPPH, ABTS and FRAP).

Regarding the antibacterial and antifungal activities, LA, LL and G90 crude essential oils present very low MICs and MBC/MFC values. This can be explained because some bioactive compounds, present in the LA, LL and G90 crude essential oils, alter the cell division. The correlation between the antibacterial and antifungal activities and the chemical composition suggested a high synergistic effect between bioactive compounds in essential oils, such as oxygenated monoterpenes (linalool, linalyl acetate, bornyl acetate and terpinen-4-ol) [59,60].

The antiproliferative potential of the LA, LL and G90 crude essential oils was evaluated in the HeLa tumoral cell line using the MTT assay. This research study about LA, LL and G90 crude essential oils on the HeLa tumoral cell line indicated a high antiproliferative potential at low concentrations, 1%, which means that the LA, LL and G90 crude essential oils contain various bioactive compounds, and each compound potentially improves or modifies the antiproliferative effects. Based on the literature, it can be inferred that the antiproliferative potential of LA, LL, and G90 crude essential oils is related to the metabolization of their chemical compounds and the synergistic effect of these compounds. Nevertheless, the antiproliferative values found are insignificant compared to those of the other lavender essential oils already tested. For instance, the lavender essential oils showed antitumoral potential in several cell lines tested (Calu-3, MCF-7, U-373, PC3) [23,24,25].

## 5. Conclusions

One of the objectives of this study was to assess the chemical composition and biological potential of a novel Romanian lavender species, *George 90*, alongside the parental lavender species *L. angustifolia* and *L. latifolia*. Lavandula genus plants have a long history of traditional medicinal use in Romania, dating back to ancient times, for treating a variety of illnesses. Through GC-MS/MS and HPLC-DAD techniques, numerous bioactive compounds from various classes were identified, each possessing significant biological properties.

The lavender species LA, LL, and G90 prove to be a rich source of bioactive compounds, offering potential applications in treating a range of human body disorders. They could serve as valuable natural resources for the nutraceutical, food, pharmaceutical and cosmetic industries. To the best of our knowledge, the chemical composition and the biological activities of Romanian G90 lavender species are reported for the first time. The LA, LL and G90 lavender species showed various concentrations of bioactive compounds with varying intensities of antioxidant activities in the ABTS, DPPH and FRAP assays. The LA, LL and G90 antioxidant bioactive compounds may play a vital role in antibacterial, antifungal and antiproliferative potentials.

## 6. Patents

The species George 90 has been homologated in Romania since 2017 (Registration certificate no. 4890/07.06.2017).

## Figures and Tables

**Table 1:** The extraction yield regarding obtaining essential oils from the *L. angustifolia*, *L. latifolia* and *George 90* lavender species.

Essential Oil	Inflorescence Mass (g)	Oil Volume (mL)	Oil Mass (g)	Extraction Yield (%)
**MAE**				
LA	450	5.00	3.68	0.82 ± 0.002 ^e^
LL	450	7.00	5.53	1.23 ± 0.001 ^c^
G90	450	11.00	8.98	2.00 ± 0.020 ^a^
**HD**				
LA	450	3.83	2.82	0.63 ± 0.010 ^f^
LL	450	6.30	4.98	1.11 ± 0.010 ^d^
G90	450	8.10	6.61	1.47 ± 0.002 ^b^

MAE—microwave assisted extraction; HD—hydrodistillation; LA—*L. angustifolia*; LL—*L. latifolia*; G90—*George 90*. Values are presented as means ± SD, *n* = 3 per treatment group. Data without a common superscript letter differ (*p* < 0.05), as analyzed by one-way ANOVA and the TUKEY test.

**Table 2:** The extract mass and the extraction yield for *L. angustifolia*, *L. latifolia* and *George 90* extracts using the MAE extraction method.

No. Crt.	Grinding Degree	Solvent Used	Solvent Report (%)	Plant Material/ Solvent Ratio	Extract Mass (g)	Extraction Yield (%)
***L. angustifolia***						
1	G1	EtOH	50	1:20	0.076 ± 0.006	5.05 ± 0.40 ^f^
2	G2	EtOH	50	1:20	0.110 ± 0.020	7.33 ± 1.37 ^ef^
3	G1	EtOH	50	1:40	0.100 ± 0.008	6.67 ± 0.52 ^ef^
4	G2	EtOH	50	1:40	0.120 ± 0.026	8.00 ± 1.73 ^ef^
5	G1	EtOH	70	1:20	0.211 ± 0.018	14.04 ± 1.18 ^acd^
6	G2	EtOH	70	1:20	0.225 ± 0.021	15.01 ± 1.38 ^ac^
7	G1	EtOH	70	1:40	0.245 ± 0.011	16.30 ± 0.70 ^ab^
8	G2	EtOH	70	1:40	0.259 ± 0.007	17.28 ± 0.46 ^a^
9	G1	MeOH	50	1:20	0.150 ± 0.006	10.03 ± 0.41 ^cf^
10	G2	MeOH	50	1:20	0.128 ± 0.029	8.55 ± 1.96 ^def^
11	G1	MeOH	50	1:40	0.167 ± 0.016	11.12 ± 1.06 ^bce^
12	G2	MeOH	50	1:40	0.155 ± 0.005	10.33 ± 0.32 ^cf^
***L. latifolia***						
1	G1	EtOH	50	1:20	0.097 ± 0.011	6.48 ± 0.76 ^d^
2	G2	EtOH	50	1:20	0.135 ± 0.010	8.98 ± 0.67 ^bcd^
3	G1	EtOH	50	1:40	0.132 ± 0.004	8.79 ± 0.26 ^cd^
4	G2	EtOH	50	1:40	0.149 ± 0.010	9.96 ± 0.64 ^bc^
5	G1	EtOH	70	1:20	0.166 ± 0.016	11.04 ± 1.03 ^ac^
6	G2	EtOH	70	1:20	0.173 ± 0.011	11.52 ± 0.74 ^ac^
7	G1	EtOH	70	1:40	0.181 ± 0.005	12.04 ± 0.36 ^ab^
8	G2	EtOH	70	1:40	0.212 ± 0.005	14.11 ± 0.32 ^a^
9	G1	MeOH	50	1:20	0.160 ± 0.010	10.64 ± 0.69 ^bc^
10	G2	MeOH	50	1:20	0.149 ± 0.002	9.96 ± 0.13 ^bc^
11	G1	MeOH	50	1:40	0.170 ± 0.006	11.32 ± 0.39 ^ac^
12	G2	MeOH	50	1:40	0.168 ± 0.010	11.19 ± 0.63 ^ac^
***George 90***						
1	G1	EtOH	50	1:20	0.130 ± 0.012	8.67 ± 0.80 ^e^
2	G2	EtOH	50	1:20	0.150 ± 0.017	10.00 ± 1.16 ^e^
3	G1	EtOH	50	1:40	0.157 ± 0.026	10.44 ± 1.77 ^e^
4	G2	EtOH	50	1:40	0.180 ± 0.002	12.02 ± 0.12 ^de^
5	G1	EtOH	70	1:20	0.274 ± 0.011	18.27 ± 0.75 ^abc^
6	G2	EtOH	70	1:20	0.298 ± 0.018	19.83 ± 1.21 ^a^
7	G1	EtOH	70	1:40	0.279 ± 0.011	18.62 ± 0.77 ^ab^
8	G2	EtOH	70	1:40	0.334 ± 0.032	22.26 ± 2.10 ^a^
9	G1	MeOH	50	1:20	0.186 ± 0.003	12.39 ± 0.21 ^cde^
10	G2	MeOH	50	1:20	0.178 ± 0.016	11.88 ± 1.06 ^de^
11	G1	MeOH	50	1:40	0.265 ± 0.003	17.68 ± 0.20 ^ad^
12	G2	MeOH	50	1:40	0.200 ± 0.030	13.34 ± 2.03 ^bde^

G1—plant material of a coarse size; G2—plant material of a very small size; EtOH—ethanol; MeOH—methanol; MAE—microwave-assisted extraction. The results are the mean of the three determinations carried out ± the standard deviation. Values are presented as means ± SD, *n* = 3 per treatment group. Data in the Extraction yield (%) column without a common superscript letter differ (*p* < 0.05), as analyzed by one-way ANOVA and the TUKEY test, for each plant species.

**Table 3:** Chemical composition of *L. angustifolia*, *L. latifolia* and *George 90* essential oils.

RT	Chemical Compound Name	Concentration (%)					
		LA-MAE	LA-HD	LL-MAE	LL-HD	G90-MAE	G90-HD
13.45	Tricyclene	0.02	0.03	0.01	0.04	0.02	0.03
13.61	2-Thujene	0.06	0.09	0.09	0.02	0.06	0.09
13.87	a-Pinene	0.73	0.97	0.17	0.13	0.20	0.15
14.47	Camphene	0.49	0.49	0.07	0.36	0.07	0.38
15.32	a-Phelandrene	0.22	0.30	0.04	0.02	0.04	0.05
15.49	2-(10)-Pinene	0.95	1.06	0.06	0.04	0.07	0.04
15.92	Myrcene	0.19	0.30	0.76	0.57	0.81	0.51
16.60	3-Carene	0.22	0.43	0.10	0.01	0.13	0.05
17.17	o-Cymene	0.17	0.09	0.13	0.65	0.15	0.68
17.31	D-Limonene	0.37	0.77	0.68	0.14	0.75	0.28
17.41	Eucalyptol	19.51	14.59	3.35	0.28	3.40	0.31
17.50	8-Terpinene	0.42	1.50	1.63	0.21	1.65	0.23
18.71	cis-Linalool oxide	0.96	0.31	0.26	4.31	0.26	4.38
19.22	trans-Linalool oxide	0.80	0.29	0.27	4.14	0.28	4.35
19.68	a-Linalool	40.38	39.10	29.97	27.78	30.00	28.12
21.19	Camphor	14.35	11.30	1.32	0.31	15.25	11.51
21.65	Lavandulol	0.65	0.69	0.40	1.37	0.60	1.40
21.99	Borneol	6.42	5.43	0.98	3.18	1.04	3.25
22.23	Terpinen-4-ol	2.73	3.44	4.80	4.30	5.00	4.43
22.42	Hexyl butyrate	0.62	0.49	0.41	1.17	0.61	1.37
22.66	a-Terpineol	1.45	0.94	3.84	2.96	3.99	3.95
24.12	Linalyl acetate	5.41	6.40	43.78	22.66	45.58	24.96
25.07	2-Isopropenyl-5-methyl-4-hexyl_acetate	1.07	0.95	3.85	5.82	3.99	6.02
25.21	Bornyl acetate	0.06	0.01	0.88	0.08	0.90	0.12
27.14	Nerol acetate	0.07	0.03	0.88	0.84	1.00	0.97
27.69	Geranyl acetate	0.08	0.03	1.51	1.13	1.71	1.33
27.86	n-Hexyl hexanoate	0.06	0.26	0.04	0.08	0.05	0.09
28.01	trans-a-Bergamotene	0.05	0.19	0.04	0.07	0.05	0.07
28.39	a-Curcumene	0.04	0.22	0.02	0.00	0.05	0.10
28.65	a-Chemigrene	0.05	0.07	0.00	0.02	0.05	0.05
28.99	Caryophyllene	0.94	4.23	3.63	0.98	4.00	1.01
29.63	a-Famesene	0.32	2.42	1.44	0.81	1.52	0.91
29.90	Humulene	0.06	0.14	0.14	0.09	0.17	0.10
30.29	a-Copaene	0.01	0.11	0.01	0.00	0.02	0.01
30.54	Germacrene D	0.02	0.16	0.31	0.08	0.42	0.12
30.82	Geranyl 2-methyl butyrate	0.22	0.40	0.00	0.01	0.01	0.01
31.30	c-Cadinene	0.02	0.09	0.03	0.13	0.04	0.15
31.40	Teresantalol	0.00	0.01	0.07	0.49	0.08	0.50
33.03	Caryophyllene oxide	0.92	0.32	7.30	0.50	7.34	0.59
34.35	t-Cadinol	0.15	0.07	0.04	1.02	0.09	1.21
35.04	Isoaromandene epoxyde	0.04	0.00	0.01	0.68	0.02	0.71

RT—retention time. The mean retention time (RT) error for the identified compounds was ±0.001–0.1 min.

**Table 4:** Identification and quantification of polyphenolic compounds present in *L. angustifolia* hydroalcoholic extracts.

RF	LA Hydroalcoholic Extracts (mg/kg Plant)											
	LA-1	LA-2	LA-3	LA-4	LA-5	LA-6	LA-7	LA-8	LA-9	LA-10	LA-11	LA-12
**TA**	28.0 ± 0.4 ^g^	34.5 ± 2.4 ^g^	49.5 ± 0.4 ^f^	50.4 ± 2.5 ^f^	83.1 ± 0.9 ^d^	91.8 ± 2.5 ^c^	111.5 ± 2.3 ^b^	122.6 ± 0.7 ^a^	56.1 ± 0.6 ^f^	51.1 ± 0.3 ^f^	74.7 ± 1.0 ^e^	69.3 ± 1.2 ^e^
**GA**	ND	ND	10 ± 0.0 ^g^	10.7 ± 0.2 ^g^	25.5 ± 0.0 ^c^	28.6 ± 0.9 ^b^	29.6 ± 0.2 ^b^	42.1 ± 0.1 ^a^	16.8 ± 0.9 ^e^	14.3 ± 0.4 ^f^	23.4 ± 0.2 ^d^	18.8 ± 0.1 ^e^
**PA**	3107.0 ± 17.0 ^k^	4350.2 ± 40.1 ^j^	4982.8 ± 48.9 ^i^	5648.7 ± 64.7 ^h^	9196.7 ± 32.1 ^d^	9857.3 ± 97.9 ^c^	10,223.0 ± 31.2 ^b^	11,025.0 ± 39.0 ^a^	7510.4 ± 21.2 ^f^	7202.9 ± 48.8 ^g^	7837.8 ± 85.7 ^e^	7793.2 ± 96.6 ^ef^
**CA**	47.1 ± 0.2 ^f^	65.9 ± 4.2 ^f^	96.5 ± 0.3 ^e^	103.5 ± 4.3 ^e^	154.8 ± 11.6 ^bc^	159.9 ± 0.5 ^bc^	172.4 ± 3.9 ^b^	221.7 ± 12.5 ^a^	117.5 ± 0.3 ^de^	105.5 ± 4.6 ^e^	137.0 ± 2.5 ^cd^	134.8 ± 0.5 ^cd^
**CF**	33.3 ± 1.2 ^g^	36.3 ± 1.2 ^fg^	41.9 ± 3.7 ^ef^	45.2 ± 2.0 ^de^	56.0 ± 0.9 ^bc^	60.5 ± 1.4 ^ab^	66.3 ± 0.3 ^a^	68.6 ± 0.9 ^a^	47.7 ± 1.2 ^ce^	46.5 ± 1.8 ^de^	55.1 ± 0.9 ^bc^	50.5 ± 1.9 ^cd^
**CH**	ND	ND	ND	ND	33.0 ± 3.8 ^b^	40.3 ± 0.9 ^b^	67.3 ± 2.1 ^a^	70.9 ± 9.4 ^a^	ND	ND	ND	ND
**SY**	1017.3 ± 11.6 ^i^	1059.0 ± 0.4 ^hi^	1180.8 ± 2.0 ^gh^	1214.5 ± 6.0 ^g^	3014.7 ± 2.0 ^c^	3181.2 ± 31.5 ^b^	3048.7 ± 38.2 ^c^	3847.3 ± 2.0 ^a^	1653.5 ± 10.8 ^e^	1426.2 ± 40.3 ^f^	1863.7 ± 49.6 ^d^	1663.7 ± 21.8 ^e^
**EP**	ND	ND	ND	ND	ND	ND	ND	ND	ND	ND	ND	ND
**PC**	10.8 ± 0.3 ^g^	14.1 ± 0.4 ^fg^	14.7 ± 1.1 ^f^	19.7 ± 1.1 ^e^	24.5 ± 0.3 ^cd^	25.6 ± 0.2 ^c^	32.3 ± 0.2 ^b^	38.1 ± 1.3 ^a^	21.4 ± 0.6 ^de^	20.8 ± 0.8 ^de^	23.4 ± 1.1 ^ce^	22.3 ± 0.5 ^ce^
**FA**	125.2 ± 5.9 ^h^	141.6 ± 7.4 ^gh^	147.4 ± 7.7 ^fh^	162.4 ± 12.0 ^efg^	194.8 ± 1.1 ^cd^	206.7 ± 0.2 ^bc^	229.4 ± 1.8 ^b^	296.0 ± 1.1 ^a^	170.2 ± 5.0 ^defg^	163.3 ± 6.2 ^efg^	178.7 ± 1.3 ^ce^	173.0 ± 6.5 ^def^
**OC**	6.1 ± 0.1 ^f^	10.8 ± 0.3 ^e^	10.9 ± 0.0 ^e^	11.6 ± 0.2 ^de^	22.3 ± 0.5 ^c^	25.6 ± 0.2 ^b^	39.4 ± 0.9 ^a^	41.1 ± 1.1 ^a^	14.7 ± 1.1 ^d^	13.0 ± 0.6 ^de^	21.4 ± 0.6 ^c^	20.8 ± 0.8 ^c^
**EA**	3598.7 ± 9.69 ^i^	4687.2 ± 39.5 ^h^	6997.6 ± 121.5 ^g^	7692.1 ± 294.2 ^fg^	9262.1 ± 172.9 ^ce^	10,021.9 ± 331.6 ^c^	12,528.9 ± 105.7 ^b^	14,443.8 ± 202.6 ^a^	8461.4 ± 300.2 ^def^	8296.8 ± 253.4 ^ef^	9325.3 ± 12.8 ^cd^	9109.3 ± 144.2 ^ce^
**IS**	972.6 ± 14.3 ^f^	1313.3 ± 25.3 ^e^	1355.2 ± 16.0 ^de^	1464.2 ± 11.8 ^ce^	2099.4 ± 29.1 ^b^	2175.8 ± 170.1 ^b^	2407.5 ± 68.1 ^b^	2776.2 ± 16.0 ^a^	1658.9 ± 0.0 ^cd^	1526.14 ± 25.8 ^ce^	1760.7 ± 108.7 ^c^	1689.1 ± 3.0 ^c^
**RT**	587.7 ± 6.7 ^g^	600.5 ± 32.9 ^g^	781.3 ± 54.9 ^f^	809.1 ± 52.6 ^ef^	1161.6 ± 22.6 ^bc^	1189.2 ± 22.9 ^bc^	1332.4 ± 38.7 ^ab^	1449.0 ± 61.4 ^a^	961.0 ± 0.8 ^de^	854.5 ± 5.4 ^ef^	1103.9 ± 34.7 ^cd^	1036.5 ± 8.1 ^cd^
**RS**	7782.0 ± 129.0 ^i^	9776.1 ± 233.9 ^h^	11,368.9 ± 124.6 ^g^	11,776.2 ± 233.7 ^fg^	19,539.2 ± 300.3 ^c^	20,700.4 ± 363.7 ^b^	29,365.8 ± 394.6 ^a^	30,181.1 ± 167.3 ^a^	12,626.8 ± 116.8 ^f^	16,352.9 ± 112.6 ^e^	19,293.1 ± 176.9 ^c^	17,664.9 ± 19.2 ^d^
**HY**	1011.6 ± 76.0 ^h^	1443.2 ± 45.2 ^gh^	1910.8 ± 78.9 ^fg^	2056.6 ± 266.8 ^eg^	3927.3 ± 96.3 ^c^	4072.3 ± 108.6 ^c^	4988.2 ± 290.9 ^b^	5733.0 ± 166.9 ^a^	2685.3 ± 108.6 ^de^	2479.4 ± 99.1 ^def^	2944.75 ± 84.7 ^d^	2455.1 ± 86.3 ^def^
**NA**	885.0 ± 16.8 ^h^	1514.2 ± 19.9 ^g^	1628.2 ± 37.6 ^g^	2305.6 ± 16.8 ^f^	4608.0 ± 153.6 ^c^	4842.8 ± 40.2 ^c^	5405.2 ± 46.8 ^b^	6760.3 ± 47.6 ^a^	3618.2 ± 24.6 ^e^	2541.2 ± 23.9 ^f^	4055.9 ± 150.8 ^d^	3724.8 ± 10.2 ^de^
**QE**	ND	ND	1526.0 ± 51.0 ^h^	1626.4 ± 34.9 ^h^	7366.8 ± 53.0 ^c^	7535.8 ± 31.9 ^c^	9471.2 ± 85.9 ^b^	9912.0 ± 123.0 ^a^	2575.7 ± 15.9 ^f^	2067.0 ± 19.4 ^g^	5417.6 ± 39.7 ^d^	4609.8 ± 19.8 ^e^
**LU**	ND	ND	ND	ND	228.3 ± 2.1 ^c^	378.8 ± 0.4 ^b^	408.1 ± 0.3 ^a^	408.7 ± 14.1 ^a^	140.8 ± 3.3 ^de^	115.7 ± 3.4 ^e^	168.0 ± 4.3 ^d^	153.8 ± 10.2 ^d^
**NR**	ND	ND	ND	ND	80.5 ± 1.6 ^c^	89.1 ± 3.9 ^bc^	100.6 ± 1.7 ^ab^	102.1 ± 2.7 ^a^	36.2 ± 1.7 ^e^	29.9 ± 0.0 ^e^	61.8 ± 3.0 ^d^	52.8 ± 5.4 ^d^
**KA**	ND	ND	ND	ND	11,345.6 ± 62.0 ^d^	11,491.2 ± 12.3 ^c^	17,106.9 ± 56.9 ^b^	20,582.8 ± 19.6 ^a^	ND	ND	ND	ND

RF—reference compounds. LA—*L. angustifolia* hydroalcoholic extracts. ND—unidentified polyphenolic compound. TA—tannic acid; GA—gallic acid; PA—protocatechuic acid; CA—catechin; CF—caffeic acid; CH—chlorogenic acid; SY—syringic acid; EP—epicatechin; PC—p-coumaric acid; FA—ferulic acid; OC—o-coumaric acid; EA—ellagic acid; IS—isoquercetin; RT—rutin; RS—rosmarinic acid; HY—hyperoside; NA—naringin; QE—quercetin; LU—luteolin; NR—naringenin; KA—kaempferol. Values are presented as means ± SD, *n* = 3 per treatment group. Data without a common superscript letter differ (*p* < 0.05), as analyzed by one-way ANOVA and the TUKEY test.

**Table 5:** Identification and quantification of polyphenolic compounds present in *L. latifolia* hydroalcoholic extracts.

RF	LL Hydroalcoholic Extracts (mg/kg Plant)											
	LL-1	LL-2	LL-3	LL-4	LL-5	LL-6	LL-7	LL-8	LL-9	LL-10	LL-11	LL-12
**TA**	28.2 ± 0.9 ^e^	34.5 ± 1.6 ^de^	40.8 ± 2.8 ^ce^	43.9 ± 2.5 ^ce^	61.2 ± 2.9 ^bc^	69.6 ± 4.0 ^b^	95.4 ± 4.3 ^a^	97.3 ± 5.5 ^a^	47.1 ± 3.3 ^be^	45.5 ± 0.6 ^be^	59.2 ± 2.5 ^bcd^	50.3 ± 14.0 ^be^
**GA**	7.0 ± 0.1 ^f^	7.3 ± 0.1 ^ef^	7.5 ± 0.2 ^ef^	8.8 ± 0.3 ^e^	12.3 ± 0.2 ^bd^	13.1 ± 0.4 ^bc^	13.7 ± 0.2 ^b^	16.5 ± 0.1 ^a^	11.7 ± 0.2 ^cd^	10.9 ± 0.6 ^d^	13.0 ± 0.7 ^bc^	12.4 ± 0.3 ^bd^
**PA**	1234.1 ± 40.1 ^f^	1659.4 ± 5.0 ^e^	1945.6 ± 10.9 ^e^	3033.5 ± 32.1 ^d^	3945.2 ± 34.4 ^b^	4027.9 ± 87.5 ^b^	6879.5 ± 125.4 ^a^	6984.7 ± 125.9 ^a^	3562.3 ± 56.2 ^c^	3340.0 ± 59.5 ^cd^	3936.3 ± 32.3 ^b^	3573.4 ± 40.5 ^c^
**CA**	103.5 ± 10.7 ^g^	151.8 ± 3.6 ^fg^	152.6 ± 20.8 ^fg^	164.2 ± 19.0 ^f^	263.8 ± 4.5 ^cd^	297.0 ± 2.4 ^ac^	320.7 ± 6.2 ^ab^	345.5 ± 1.8 ^a^	175.4 ± 0.5 ^ef^	166.8 ± 6.7 ^f^	278.3 ± 13.9 ^bc^	220.8 ± 8.7 ^de^
**CF**	59.4 ± 4.6 ^f^	69.5 ± 0.4 ^f^	76.1 ± 3.8 ^ef^	88.4 ± 4.4 ^ef^	196.7 ± 1.4 ^b^	215.3 ± 27.1 ^ab^	229.8 ± 4.7 ^ab^	248.1 ± 5.0 ^a^	116.5 ± 0.0 ^ce^	95.0 ± 1.7 ^def^	139.5 ± 4.1 ^c^	134.6 ± 2.4 ^cd^
**CH**	87.2 ± 3.2 ^f^	96.4 ± 0.5 ^ef^	103.0 ± 2.9 ^df^	103.0 ± 2.9 ^df^	123.3 ± 0.7 ^abc^	128.8 ± 5.0 ^abc^	130.9 ± 0.1 ^ab^	139.4 ± 1.4 ^a^	112.8 ± 5.1 ^cde^	103.3 ± 1.9 ^df^	120.0 ± 3.5 ^bd^	117.4 ± 6.7 ^bd^
**SY**	302.4 ± 3.4 ^h^	526.6 ± 4.5 ^g^	893.4 ± 3.5 ^f^	1041.6 ± 23.6 ^ef^	1537.9 ± 23.7 ^c^	1562.4 ± 34.2 ^c^	1814.1 ± 25.9 ^b^	2023.9 ± 7.5 ^a^	1340.4 ± 64.9 ^d^	1120.4 ± 31.8 ^e^	1558.2 ± 42.3 ^c^	1449.0 ± 28.6 ^cd^
**EP**	ND	60.7 ± 4.3 ^f^	119.6 ± 0.6 ^e^	156.3 ± 1.0 ^de^	261.3 ± 1.5 ^b^	281.8 ± 2.5 ^b^	295.8 ± 2.1 ^b^	395.1 ± 15.7 ^a^	168.1 ± 4.4 ^cd^	151.0 ± 1.4 ^de^	202.7 ± 19.9 ^c^	173.6 ± 11.5 ^cd^
**PC**	39.7 ± 0.6 ^f^	52.1 ± 0.0 ^f^	57.1 ± 1.4 ^f^	65.1 ± 0.7 ^ef^	112.9 ± 4.7 ^cd^	127.3 ± 7.3 ^bc^	146.6 ± 7.4 ^ab^	156.5 ± 8.3 ^a^	92.1 ± 0.7 ^d^	90.5 ± 8.7 ^de^	104.2 ± 3.0 ^cd^	100.8 ± 3.5 ^d^
**FA**	151.3 ± 6.8 ^g^	210.0 ± 24.1 ^fg^	239.1 ± 7.2 ^fg^	248.1 ± 2.0 ^fg^	489.3 ± 5.2 ^bd^	509.9 ± 43.8 ^bc^	568.8 ± 16.8 ^ab^	622.2 ± 46.7 ^a^	291.2 ± 4.0 ^ef^	272.4 ± 8.5 ^f^	410.4 ± 9.7 ^cd^	382.9 ± 3.4 ^de^
**OC**	7.5 ± 0.2 ^e^	16.1 ± 1.4 ^de^	30.6 ± 1.2 ^cd^	44.9 ± 3.7 ^bc^	53.7 ± 3.3 ^b^	55.7 ± 2.6 ^b^	78.8 ± 8.2 ^a^	79.8 ± 1.2 ^a^	49.4 ± 1.3 ^b^	47.4 ± 3.0 ^b^	54.3 ± 0.1 ^b^	53.7 ± 1.0 ^b^
**EA**	5642.8 ± 4.0 ^i^	7244.9 ± 121.6 ^h^	7655.6 ± 188.7 ^h^	9468.2 ± 19.6 ^g^	16,189.5 ± 181.4 ^cd^	17,389.9 ± 176.0 ^bc^	17,861.0 ± 154.2 ^b^	19,572.4 ± 480.3 ^a^	11,639.6 ± 282.5 ^f^	10,480.3 ± 294.9 ^fg^	15,547.2 ± 348.2 ^d^	13,113.9 ± 204.7 ^e^
**IS**	811.7 ± 8.2 ^f^	996.5 ± 9.0 ^e^	1254.5 ± 70.5 ^d^	1322.3 ± 29.0 ^d^	1817.7 ± 76.0 ^ac^	1858.6 ± 28.6 ^ab^	1914.6 ± 13.6 ^a^	1950.0 ± 23.5 ^a^	1728.5 ± 12.9 ^bc^	1661.1 ± 9.0 ^c^	1949.9 ± 14.1 ^a^	1735.0 ± 14.3 ^bc^
**RT**	162.2 ± 2.1 ^g^	179.2 ± 0.9 ^g^	265.8 ± 19.0 ^f^	307.1 ± 21.4 ^ef^	615.2 ± 0.3 ^c^	636.9 ± 0.4 ^c^	688.6 ± 6.6 ^b^	815.8 ± 13.8 ^a^	343.5 ± 11.0 ^e^	308.8 ± 13.4 ^ef^	484.2 ± 3.8 ^d^	464.1 ± 7.1 ^d^
**RS**	10,733.0 ± 33.0 ^j^	16,950.2 ± 49.8 ^i^	21,590.4 ± 35.2 ^h^	21,797.8 ± 79.7 ^h^	30,411.3 ± 31.4 ^f^	34,181.0.8 ± 44.8 ^e^	36,134.7 ± 74.9 ^c^	39,841.0 ± 21.0 ^a^	34,159.8 ± 174.6 ^e^	24,298.9 ± 98.9 ^g^	38,892.1 ± 41.8 ^b^	35,692.0 ± 18.7 ^d^
**HY**	632.3 ± 5.0 ^g^	1445.0 ± 6.0 ^f^	1851.4 ± 25.8 ^e^	2142.7 ± 88.3 ^de^	2632.2 ± 35.1 ^b^	2764.9 ± 48.6 ^b^	3235.1 ± 195.7 ^a^	3429.1 ± 109.1 ^a^	2492.6 ± 19.3 ^bd^	2191.1 ± 41.5 ^cde^	2564.3 ± 59.9 ^bc^	2512.2 ± 48.7 ^bd^
**NA**	1606.0 ± 92.0 ^i^	1815.0 ± 48.8 ^i^	2633.2 ± 22.9 ^h^	3321.0 ± 54.9 ^g^	4878.7 ± 134.6 ^d^	6992.8 ± 39.9 ^c^	7621.0 ± 48.8 ^b^	9162.3 ± 28.2 ^a^	4421.3 ± 64.9 ^e^	4038.9 ± 94.6 ^f^	4977.9 ± 78.9 ^d^	4522.6 ± 22.7 ^e^
**QE**	ND	ND	748.8 ± 2.48 ^f^	1212.7 ± 17.8 ^e^	3753.6 ± 164.8 ^c^	4365.6 ± 189.9 ^b^	4411.5 ± 15.9 ^ab^	4775.9 ± 7.9 ^a^	2771.2 ± 35.8 ^d^	2751.0 ± 22.8 ^d^	3629.2 ± 62.4 ^c^	2827.8 ± 7.2 ^d^
**LU**	43.0 ± 0.4 ^g^	50.0 ± 1.9 ^g^	71.9 ± 2.2 ^f^	73.4 ± 1.9 ^f^	138.9 ± 1.3 ^b^	126.8 ± 2.9 ^bc^	188.9 ± 2.3 ^a^	203.5 ± 3.6 ^a^	96.5 ± 1.5 ^de^	86.5 ± 0.7 ^ef^	116.3 ± 9.9 ^cd^	101.2 ± 7.1 ^de^
**NR**	50.5 ± 7.0 ^g^	74.2 ± 0.8 ^f^	87.1 ± 2.7 ^ef^	102.8 ± 2.5 ^de^	128.2 ± 3.4 ^bc^	134.4 ± 4.3 ^b^	143.0 ± 4.4 ^b^	164.4 ± 4.2 ^a^	109.2 ± 1.2 ^cd^	102.8 ± 3.3 ^de^	134.1 ± 6.5 ^b^	129.2 ± 2.4 ^bc^
**KA**	ND	ND	ND	ND	ND	ND	ND	ND	ND	ND	ND	ND

RF—reference compounds. LL—*L. latifolia* hydroalcoholic extracts. ND—unidentified polyphenolic compound. TA—tannic acid; GA—gallic acid; PA—protocatechuic acid; CA—catechin; CF—caffeic acid; CH—chlorogenic acid; SY—syringic acid; EP—epicatechin; PC—p-coumaric acid; FA—ferulic acid; OC—o-coumaric acid; EA—ellagic acid; IS—isoquercetin; RT—rutin; RS—rosmarinic acid; HY—hyperoside; NA—naringin; QE—quercetin; LU—luteolin; NR—naringenin; KA—kaempferol. Values are presented as means ± SD, *n* = 3 per treatment group. Data without a common superscript letter differ (*p* < 0.05), as analyzed by one-way ANOVA and the TUKEY test.

**Table 6:** Identification and quantification of polyphenolic compounds present in *George 90* hydroalcoholic extracts.

RF	G90 Hydroalcoholic Extracts (mg/kg Plant)											
	G90-1	G90-2	G90-3	G90-4	G90-5	G90-6	G90-7	G90-8	G90-9	G90-10	G90-11	G90-12
**TA**	30.9 ± 3.3 ^g^	36.3 ± 1.0 ^g^	49.4 ± 2.4 ^f^	50.4 ± 1.5 ^f^	100.1 ± 2.9 ^cd^	109.5 ± 3.5 ^c^	123.6 ± 1.3 ^b^	142.6 ± 1.7 ^a^	56.6 ± 1.4 ^f^	51.9 ± 0.9 ^f^	94.4 ± 1.3 ^d^	69.3 ± 0.9 ^e^
**GA**	7.5 ± 0.6 ^g^	7.8 ± 1.0 ^g^	11.0 ± 0.5 ^fg^	13.9 ± 0.6 ^f^	35.7 ± 0.7 ^cd^	38.5 ± 1.1 ^bc^	41.6 ± 1.0 ^b^	49.1 ± 1.2 ^a^	26.7 ± 1.3 ^e^	24.5 ± 1.4 ^e^	33.3 ± 1.2 ^d^	38.6 ± 0.8 ^bc^
**PA**	6117.1 ± 33.7 ^j^	6349.8 ± 29.7 ^i^	6994.8 ± 35.8 ^h^	7348.8 ± 52.7 ^g^	10,946.8 ± 22.8 ^c^	10,256.8 ± 81.2 ^d^	12,123.8 ± 10.9 ^b^	13,065.3 ± 20.2 ^a^	9621.2 ± 21.1 ^ef^	9412.8 ± 41.8 ^f^	9637.8 ± 66.7 ^ef^	9783.8 ± 67.0 ^e^
**CA**	207.5 ± 18.0 ^e^	262.9 ± 2.6 ^de^	272.8 ± 18.9 ^de^	284.6 ± 38.7 ^de^	371.5 ± 5.9 ^abc^	399.0 ± 0.6 ^ab^	429.7 ± 5.2 ^a^	445.0 ± 2.8 ^a^	335.5 ± 1.5 ^bd^	306.8 ± 16.7 ^cd^	388.5 ± 17.8 ^ab^	340.5 ± 9.7 ^bd^
**CF**	70.9 ± 2.6 ^h^	99.2 ± 1.4 ^gh^	116.1 ± 1.8 ^fg^	188.0 ± 3.4 ^cd^	206.3 ± 2.0 ^c^	275.6 ± 16.7 ^b^	291.5 ± 2.7 ^ab^	308.4 ± 4.0 ^a^	146.5 ± 3.0 ^ef^	135.0 ± 9.9 ^ef^	219.1 ± 5.2 ^c^	164.2 ± 4.1 ^de^
**CH**	98.1 ± 1.2 ^f^	100.4 ± 2.5 ^f^	121.0 ± 1.9 ^ef^	133.4 ± 3.0 ^de^	145.2 ± 1.0 ^ce^	168.2 ± 8.0 ^bc^	182.0 ± 0.9 ^b^	219.6 ± 9.1 ^a^	152.3 ± 3.1 ^cd^	133.2 ± 9.1 ^de^	160.2 ± 5.0 ^bc^	146.4 ± 1.7 ^cd^
**SY**	1118.0 ± 18.9 ^f^	1378.8 ± 41.9 ^e^	1781.3 ± 54.4 ^cd^	1874.3 ± 67.8 ^cd^	3574.0 ± 26.7 ^b^	3681.0 ± 21.3 ^b^	3747.3 ± 78.2 ^b^	4074.8 ± 100.2 ^a^	1872.9 ± 13.8 ^cd^	1695.7 ± 21.0 ^d^	1974.1 ± 39.9 ^c^	1804.0 ± 28.9 ^cd^
**EP**	ND	ND	209.9 ± 1.6 ^f^	245.2 ± 2.0 ^de^	331.7 ± 9.5 ^c^	357.8 ± 3.5 ^bc^	378.8 ± 3.1 ^b^	446.2 ± 11.0 ^a^	255.3 ± 5.4 ^de^	231.0 ± 1.4 ^ef^	275.7 ± 9.0 ^d^	243.4 ± 13.0 ^df^
**PC**	49.5 ± 3.6 ^i^	66.3 ± 2 ^hi^	86.7 ± 4 ^gh^	95.9 ± 2.3 ^fg^	132.5 ± 1.7 ^ce^	146.2 ± 5.3 ^bc^	163.5 ± 5.4 ^b^	187.5 ± 4.3 ^a^	112.9 ± 1.7 ^ef^	100.3 ± 7.2 ^fg^	136.5 ± 5.0 ^cd^	122.4 ± 4.5 ^de^
**FA**	201.4 ± 2.8 ^h^	270.2 ± 14.0 ^g^	309.3 ± 5.2 ^fg^	338.5 ± 4.0 ^f^	579.2 ± 2.2 ^c^	605.9 ± 23.9 ^c^	658.8 ± 3.7 ^b^	832.2 ± 16.7 ^a^	496.4 ± 6.0 ^d^	432.5 ± 2.5 ^e^	58.40 ± 3.7 ^c^	502.9 ± 3.4 ^d^
**OC**	27.0 ± 2.2 ^f^	36.4 ± 2.4 ^f^	53.2 ± 2.2 ^e^	64.3 ± 2.7 ^de^	69.7 ± 4.3 ^cd^	75.2 ± 1.6 ^bd^	86.4 ± 5.2 ^ab^	90.8 ± 3.2 ^a^	73.4 ± 2.3 ^bd^	67.3 ± 2.0 ^de^	83.2 ± 1.1 ^abc^	75.7 ± 2.0 ^bd^
**EA**	6743.0 ± 30.0 ^k^	7944.9 ± 28.6 ^j^	9356.0 ± 89.9 ^i^	10,067.9 ± 49.6 ^h^	16,990.0 ± 83.5 ^d^	19,450.9 ± 16.8 ^c^	20,861.8 ± 54.4 ^b^	22,528.1 ± 88.7 ^a^	14,600.2 ± 81.9 ^f^	13,450.9 ± 95.6 ^g^	16,947.4 ± 48.8 ^d^	15,714.6 ± 54.3 ^e^
**IS**	1016.3 ± 6.2 ^h^	1197.3 ± 5.0 ^g^	17,501.2 ± 30.9 ^ef^	1832.8 ± 19.9 ^e^	1997.6 ± 15.9 ^cd^	2099.8 ± 42.3 ^c^	2356.5 ± 19.2 ^b^	2550.4 ± 32.1 ^a^	1729.0 ± 12.9 ^ef^	1661.4 ± 9.0 ^f^	1949.6 ± 13.9 ^d^	1735.0 ± 14.5 ^ef^
**RT**	477.9 ± 26.8 ^e^	580.2 ± 38.9 ^e^	861.5 ± 43.2 ^d^	978.4 ± 31.8 ^d^	1491.8 ± 42.6 ^b^	1608.9 ± 36.7 ^ab^	1692.0 ± 91.3 ^ab^	1748.9 ± 71.9 ^a^	1041.2 ± 37.8 ^cd^	1253.9 ± 41.0 ^c^	1564.4 ± 15.5 ^ab^	1635.4 ± 15.0 ^ab^
**RS**	17,432.1 ± 22.0 ^j^	19,950.2 ± 49.7 ^i^	29,529.7 ± 38.7 ^h^	31,567.8 ± 80.2 ^g^	34,711.1 ± 51.3 ^e^	36,189.2 ± 52.4 ^d^	39,185.1 ± 44.6 ^b^	40,971.1 ± 114.4 ^a^	37,192.5 ± 75.6 ^c^	34,211.0 ± 99.4 ^f^	39,192.5 ± 29.3 ^b^	36,999.0 ± 89.9 ^c^
**HY**	1812.3 ± 46.2 ^i^	2042.8 ± 34.7 ^hi^	2311.0 ± 38.9 ^h^	2846.7 ± 25.6 ^fg^	4066.7 ± 55.6 ^d^	4362.0 ± 18.9 ^c^	5087.8 ± 90.9 ^b^	6592.6 ± 45.9 ^a^	2959.0 ± 68.9 ^fg^	2700.0 ± 68.9 ^g^	3245.5 ± 54.7 ^e^	3065.4 ± 62.9 ^ef^
**NA**	2009.1 ± 51.8 ^j^	2852.8 ± 72.8 ^i^	4038.9 ± 36.7 ^h^	4521.2 ± 55.3 ^g^	7792.1 ± 53.0 ^c^	8791.1 ± 29.9 ^b^	9921.4 ± 18.9 ^a^	10,061.9 ± 108.2 ^a^	5321.3 ± 45.2 ^ef^	5078.9 ± 35.0 ^f^	5987.8 ± 38.9 ^d^	5462.9 ± 38.6 ^e^
**QE**	ND	ND	2561.1 ± 19.0 ^h^	3661.0 ± 52.2 ^g^	8267.4 ± 23.0 ^b^	8562.4 ± 28.9 ^b^	9961.0 ± 95.6 ^a^	10,212.0 ± 133.9 ^a^	4735.9 ± 55.9 ^e^	4074.9 ± 90.9 ^f^	6481.0 ± 44.7 ^c^	5712.0 ± 49.8 ^d^
**LU**	83.2 ± 4.1 ^f^	95.0 ± 1.2 ^f^	101.4 ± 2.9 ^f^	114.5 ± 9.0 ^f^	398.6 ± 11.9 ^c^	478.4 ± 4.2 ^b^	506.1 ± 3.1 ^ab^	528.7 ± 10.9 ^a^	241.0 ± 12.9 ^de^	217.7 ± 4.0 ^e^	268.4 ± 3.4 ^d^	235.8 ± 17.9 ^de^
**NR**	78.5 ± 5.0 ^g^	94.4 ± 1.8 ^g^	117.1 ± 1.7 ^f^	123.6 ± 1.5 ^ef^	168.2 ± 1.4 ^bc^	174.4 ± 2.3 ^bc^	182.0 ± 2.4 ^ab^	199.2 ± 11.9 ^a^	159.9 ± 1.3 ^cd^	142.8 ± 2.3 ^de^	174.2 ± 3.5 ^bc^	169.3 ± 1.4 ^bc^
**KA**	ND	ND	ND	ND	ND	ND	ND	ND	ND	ND	ND	ND

RF—reference compounds. G90—*George 90* hydroalcoholic extracts. ND—unidentified polyphenolic compound. TA—tannic acid; GA—gallic acid; PA—protocatechuic acid; CA—catechin; CF—caffeic acid; CH—chlorogenic acid; SY—syringic acid; EP—epicatechin; PC—p-coumaric acid; FA—ferulic acid; OC—o-coumaric acid; EA—ellagic acid; IS—isoquercetin; RT—rutin; RS—rosmarinic acid; HY—hyperoside; NA—naringin; QE—quercetin; LU—luteolin; NR—naringenin; KA—kaempferol. Values are presented as means ± SD, *n* = 3 per treatment group. Data without a common superscript letter differ (*p* < 0.05), as analyzed by one-way ANOVA and the TUKEY test.

**Table 7:** MIC and MBC values of LA, LL and G90 essential oils against *Gram-positive* bacteria.

*Gram-Positive* Bacteria	LA-MAE	LA-HD	LL-MAE	LL-HD	G90-MAE	G90-HD	CIP
**MIC (%)**							**(µg/mL)**
***B. cereus***	5.0 ± 0.05 ^b^	10.0 ± 0.01 ^a^	5.0 ± 0.04 ^b^	5.0 ± 0.01 ^b^	5.0 ± 0.06 ^b^	10.0 ± 0.02 ^a^	0.3
***B. subtilis***	2.5 ± 0.02 ^b^	5.0 ± 0.02 ^a^	2.5 ± 0.03 ^b^	2.5 ± 0.03 ^b^	2.5 ± 0.02 ^b^	5.0 ± 0.05 ^a^	0.2
**MBC (%)**							**(µg/mL)**
***B. cereus***	10.0 ± 0.11	>10.0 ± 0.09	10.0 ± 0.08	10.0 ± 0.14	10.0 ± 0.03	>10.0 ± 0.05	0.3
***B. subtilis***	5.0 ± 0.01 ^b^	10.0 ± 0.12 ^a^	5.0 ± 0.04 ^b^	5.0 ± 0.07 ^b^	5.0 ± 0.06 ^b^	10.0 ± 0.13 ^a^	0.2

CIP—Ciprofloxacin. The experiments were conducted in triplicate, and the average MIC and MBC values were calculated. Values are presented as means ± SD, *n* = 3 per treatment group. Data without a common superscript letter differ (*p* < 0.05), as analyzed by one-way ANOVA and the TUKEY test.

**Table 8:** MIC and MFC values of LA, LL and G90 essential oils against *A. brasiliensis*, *F. oxysporum* and *P. expansum*.

Fungus	LA-MAE	LA-HD	LL-MAE	LL-HD	G90-MAE	G90-HD	FLC
**MIC (%)**							**(µg/mL)**
***A. brasiliensis***	1.3 ± 0.01	1.3 ± 0.04	1.3 ± 0.08	1.3 ± 0.03	1.3 ± 0.11	1.3 ± 0.02	-
***F. oxysporum***	2.5 ± 0.14	2.5 ± 0.18	2.5 ± 0.12	2.5 ± 0.09	2.5 ± 0.05	2.5 ± 0.01	100.0
***P. expansum***	40.0 ± 0.01 ^a^	40.0 ± 0.05 ^a^	40.0 ± 0.01 ^a^	40.0 ± 0.03 ^a^	20.0 ± 0.02 ^b^	40.0 ± 0.06 ^a^	-
**MFC (%)**							**(µg/mL)**
***A. brasiliensis***	1.3 ± 0.07	1.3 ± 0.10	1.3 ± 0.18	1.3 ± 0.07	1.3 ± 0.03	1.3 ± 0.01	-
***F. oxysporum***	2.5 ± 0.01	2.5 ± 0.19	2.5 ± 0.16	2.5 ± 0.01	2.5 ± 0.08	2.5 ± 0.20	100.0
***P. expansum***	40.0 ± 0.11 ^a^	40.0 ± 0.01 ^a^	40.0 ± 0.03 ^a^	40.0 ± 0.05 ^a^	20.0 ± 0.13 ^b^	40.0 ± 0.02 ^a^	-

FLC—fluconazole. The experiments were conducted in triplicate, and the average MIC and MFC values were calculated. Values are presented as means ± SD, *n* = 3 per treatment group. Data without a common superscript letter differ (*p* < 0.05), as analyzed by one-way ANOVA and the TUKEY test.

## Data Availability

Data is contained within the article and supplementary materials.

## Supplementary Materials

The following supporting information can be downloaded at: https://www.mdpi.com/article/10.3390/antiox12122127/s1, Figure S1: DPPH antioxidant potential expressed as the inhibition percent of (a) LA, LL and G90 essential oils and (b) LA, (c) LL and (d) G90 hydroalcoholic extracts. LA-MAE—*L. angustifolia* MAE essential oil, LA-HD—*L. angustifolia* HD essential oil, LL-MAE—*L. latifolia* MAE essential oil, LL-HD—*L. latifolia* HD essential oil, G90-MAE—*George 90* MAE essential oil and G90-HD—*George 90* HD essential oil. 1—EtOH 50%, plant material/solvent ratio at 1:20 (*m*/*v*), G1 coarse-sized plant material; 2—EtOH 50%, plant material/solvent ratio at 1:20 (*m*/*v*), G2 fine-sized plant material; 3—EtOH 50%, plant material/solvent ratio at 1:40 (*m*/*v*), G1 coarse-sized plant material; 4—EtOH 50%, plant material/solvent ratio at 1:40 (*m*/*v*), G2 fine-sized plant material; 5—EtOH 70%, plant material/solvent ratio at 1:20 (*m*/*v*), G1 coarse-sized plant material; 6—EtOH 70%, plant material/solvent ratio at 1:20 (*m*/*v*), G2 fine-sized plant material; 7—EtOH 70%, plant material/solvent ratio at 1:40 (*m*/*v*), G1 coarse-sized plant material; 8—EtOH 70%, plant material/solvent ratio at 1:40 (*m*/*v*), G2 fine-sized plant material; 9—MeOH 50%, plant material/solvent ratio at 1:20 (*m*/*v*), G1 coarse-sized plant material; 10—MeOH 50%, plant material/solvent ratio at 1:20 (*m*/*v*), G2 fine-sized plant material; 11—MeOH 50%, plant material/solvent ratio at 1:40 (*m*/*v*), G1 coarse-sized plant material; 12—MeOH 50%, plant material/solvent ratio at 1:40 (*m*/*v*), G2 fine-sized plant material. Values are presented as means ± SD, *n* = 3 per treatment group. Data without a common superscript letter differ (*p* < 0.05), as analyzed by one-way ANOVA and the TUKEY test. Figure S2: ABTS antioxidant potential expressed as the inhibition percent of (a) LA, LL and G90 essential oils and (b) LA, (c) LL and (d) G90 hydroalcoholic extracts. LA-MAE—*L. angustifolia* MAE essential oil, LA-HD—*L. angustifolia* HD essential oil, LL-MAE—*L. latifolia* MAE essential oil, LL-HD—*L. latifolia* HD essential oil, G90-MAE—*George 90* MAE essential oil and G90-HD—*George 90* HD essential oil. 1—EtOH 50%, plant material/solvent ratio at 1:20 (*m*/*v*), G1 coarse-sized plant material; 2—EtOH 50%, plant material/solvent ratio at 1:20 (*m*/*v*), G2 fine-sized plant material; 3—EtOH 50%, plant material/solvent ratio at 1:40 (*m*/*v*), G1 coarse-sized plant material; 4—EtOH 50%, plant material/solvent ratio at 1:40 (*m*/*v*), G2 fine-sized plant material; 5—EtOH 70%, plant material/solvent ratio at 1:20 (*m*/*v*), G1 coarse-sized plant material; 6—EtOH 70%, plant material/solvent ratio at 1:20 (*m*/*v*), G2 fine-sized plant material; 7—EtOH 70%, plant material/solvent ratio at 1:40 (*m*/*v*), G1 coarse-sized plant material; 8—EtOH 70%, plant material/solvent ratio at 1:40 (*m*/*v*), G2 fine-sized plant material; 9—MeOH 50%, plant material/solvent ratio at 1:20 (*m*/*v*), G1 coarse-sized plant material; 10—MeOH 50%, plant material/solvent ratio at 1:20 (*m*/*v*), G2 fine-sized plant material; 11—MeOH 50%, plant material/solvent ratio at 1:40 (*m*/*v*), G1 coarse-sized plant material; 12—MeOH 50%, plant material/solvent ratio at 1:40 (*m*/*v*), G2 fine-sized plant material. Values are presented as means ± SD, *n* = 3 per treatment group. Data without a common superscript letter differ (*p* < 0.05), as analyzed by one-way ANOVA and the TUKEY test. Figure S3: Cell morphological changes induced by the (a) negative control, (b) LA, (c) LL and (d) G90 essential oils.
