# Supplementary material for: Chemical Composition and Biological Properties of New Romanian Lavandula Species
Source: Antioxidants (Basel). 2023 Dec 16;12(12):2127. doi: 10.3390/antiox12122127 (PMC10741150; doi:10.3390/antiox12122127)
Supplement: Supplementary file 1 [file antioxidants-12-02127-s001.zip › antioxidants-2759409-supplementary.pdf]

Supplementary Materials

Chemical Composition and Biological Properties of New Romanian *Lavandula* species

Ionuț Georgică Marchidan [1], Alina Ortan [2,*], Simona Marcu Spinu [2], Sorin Marius Avramescu [3], Ionela Avram [4,*], Radu Claudiu Fierascu [5] and Narcisa Babeanu [1]

[1] Biotechnologies Department, Faculty of Biotechnologies, University of Agronomic Sciences and Veterinary Medicine of Bucharest, 59 Marasti Blvd, District 1, 011464 Bucharest, Romania; ionut.marchidan@biotehnologii.usamv.ro (I.G.M.); narcisa.babeanu@biotehnologii.usamv.ro (N.B.)
[2] Mathematics, Physics and Measurements Department, Faculty of Land Reclamation and Environmental Engineering, University of Agronomic Sciences and Veterinary Medicine of Bucharest, 59 Marasti Blvd, District 1, 011464 Bucharest, Romania; simona.spinu@fifim.ro (S.M.S.)
[3] Department of Inorganic Chemistry, Organic Chemistry, Biochemistry and Catalysis, Faculty of Chemistry, University of Bucharest, 90–92 Soseaua Panduri, 050663 Bucharest, Romania; sorin.avramescu@g.unibuc.ro
[4] Department of Genetics, University of Bucharest, 1-3-Aleea Portocalelor, 060101 Bucharest, Romania
[5] National Institute for Research & Development in Chemistry and Petrochemistry-ICECHIM–Bucharest, 060021 Bucharest, Romania; fierascu.radu@icechim.ro
* Correspondence: alina.ortan@fifim.ro (A.O.); ionela.avram@unibuc.ro (I.A.)

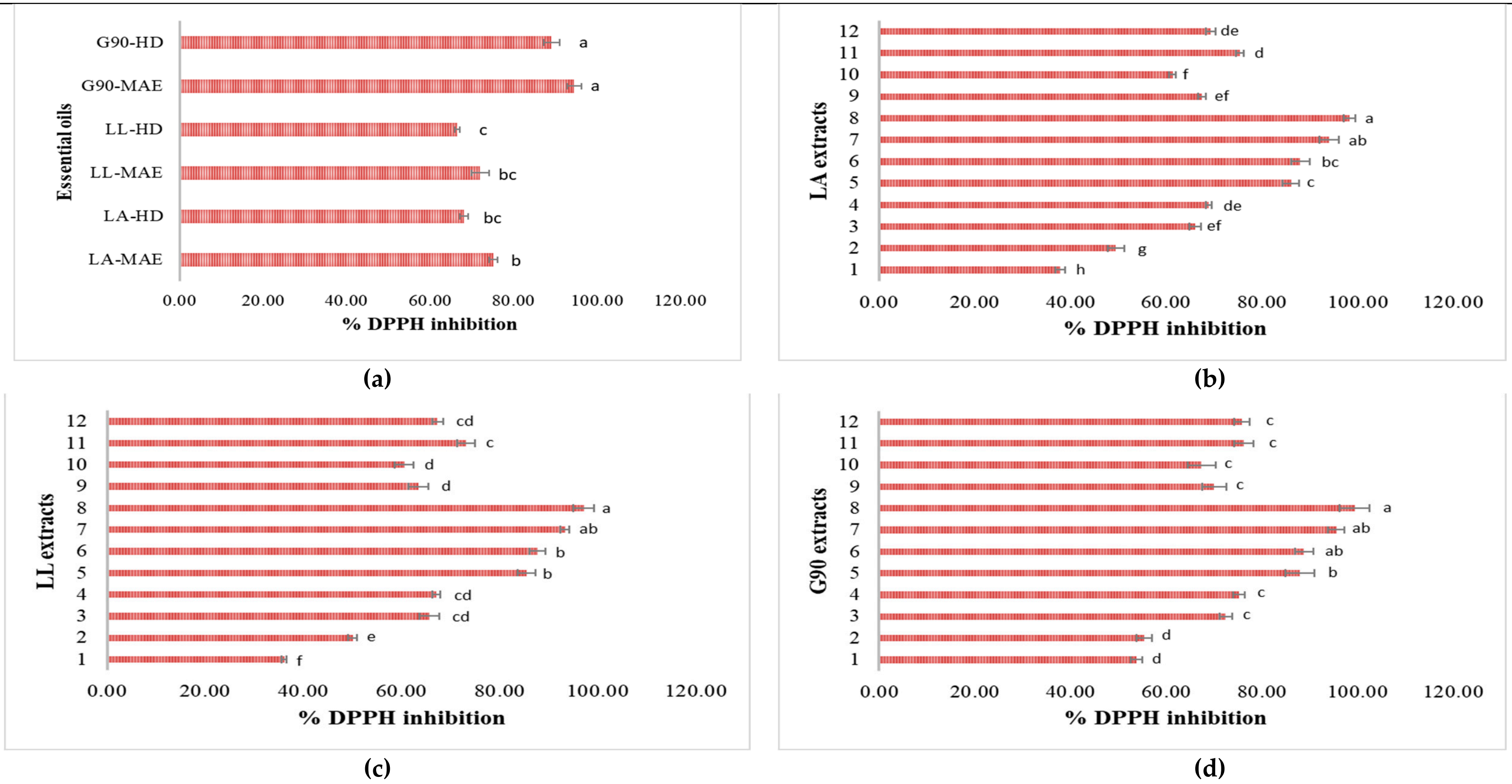

Figure S1. DPPH antioxidant potential expressed as inhibition percent of (a) LA, LL and G90 essential oils and (b) LA, (c) LL and (d) G90 hydroalcoholic extracts. LA-MAE - *L. angustigolia* MAE essential oil, LA-HD - *L. angustigolia* HD essential oil, LL-MAE - *L. latifolia* MAE essential oil, LL-HD - *L. latifolia* HD essential oil, G90-MAE - *George 90* MAE essential oil and G90-HD - *George 90* HD essential oil. 1 – EtOH 50%, plant material/solvent ratio at 1:20 (m/v), G1 coarse-sized plant material; 2 – EtOH 50%, plant material/solvent ratio at 1:20 (m/v), G2 fine-sized plant material; 3 – EtOH 50%, plant material/solvent ratio at 1:40 (m/v), G1 coarse-sized plant material; 4 – EtOH 50%, plant material/solvent ratio at 1:40 (m/v), G2 fine-sized plant material; 5 – EtOH 70%, plant material/solvent ratio at 1:20 (m/v), G1 coarse-sized plant material; 6 – EtOH 70%, plant material/solvent ratio at 1:20 (m/v), G2 fine-sized plant material; 7 – EtOH 70%, plant material/solvent ratio at 1:40 (m/v), G1 coarse-sized plant material; 8 – EtOH 70%, plant material/solvent ratio at 1:40 (m/v), G2 fine-sized plant material; 9 – MeOH 50%, plant material/solvent ratio at 1:20 (m/v), G1 coarse-sized plant material; 10 – MeOH 50%, plant material/solvent ratio at 1:20 (m/v), G2 fine-sized plant material; 11 – MeOH 50%, plant material/solvent ratio at 1:40 (m/v), G1 coarse-sized plant material; 12 – MeOH 50%, plant material/solvent ratio at 1:40 (m/v), G2 fine-sized plant material. Values are presented as means ± SD, n = 3 per treatment group. Data without a common superscript letter differ ($P < 0.05$) as analyzed by one-way ANOVA and the TUKEY test.

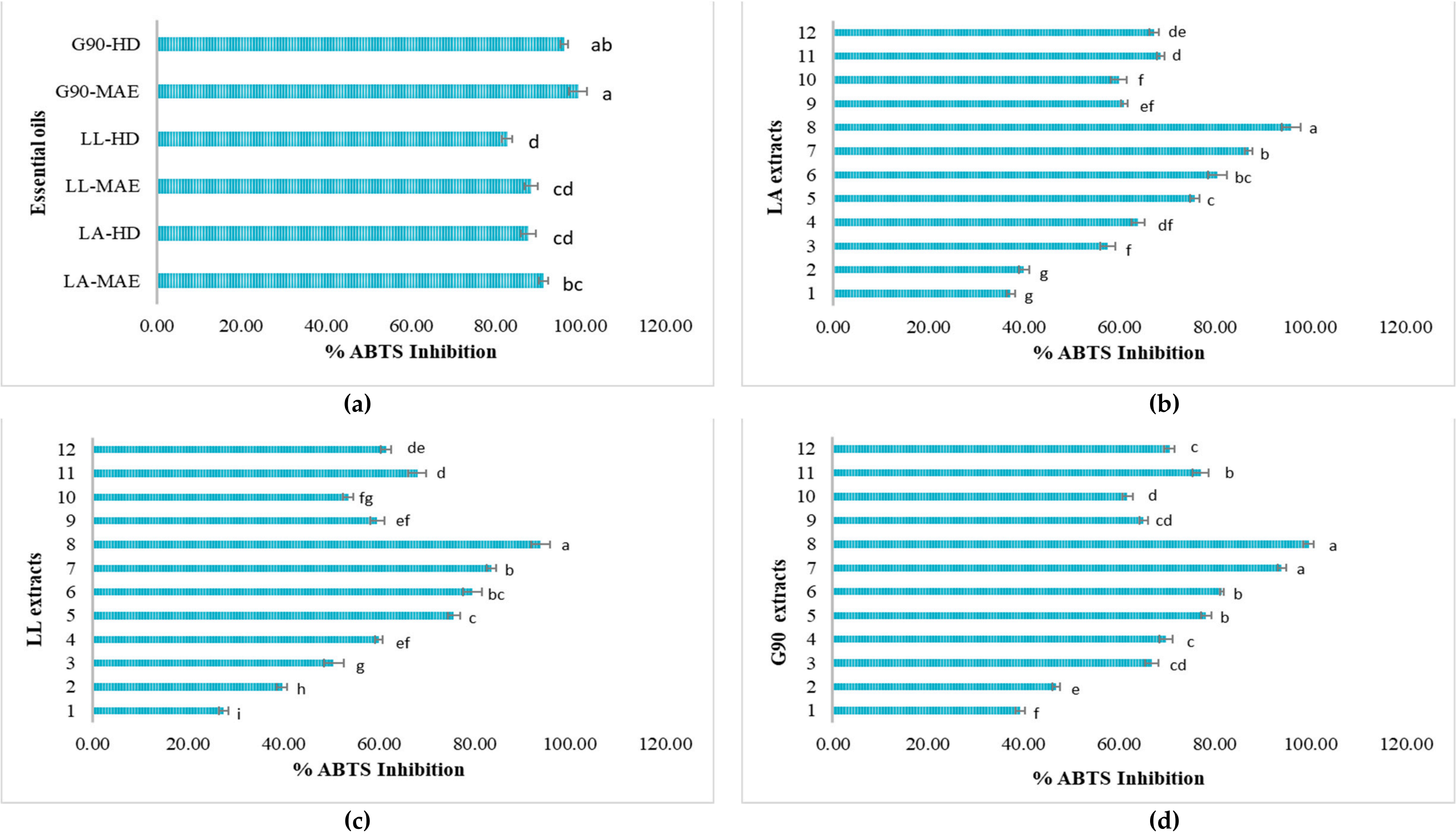

Figure S2. ABTS antioxidant potential expressed as inhibition percent of (a) LA, LL and G90 essential oils and (b) LA, (c) LL and (d) G90 hydroalcoholic extracts. LA-MAE - *L. angustigolia* MAE essential oil, LA-HD - *L. angustigolia* HD essential oil, LL-MAE - *L. latifolia* MAE essential oil, LL-HD - *L. latifolia* HD essential oil, G90-MAE - *George 90* MAE essential oil and G90-HD - *George 90* HD essential oil. 1 – EtOH 50%, plant material/solvent ratio at 1:20 (m/v), G1 coarse-sized plant material; 2 – EtOH 50%, plant material/solvent ratio at 1:20 (m/v), G2 fine-sized plant material; 3 – EtOH 50%, plant material/solvent ratio at 1:40 (m/v), G1 coarse-sized plant material; 4 – EtOH 50%, plant material/solvent ratio at 1:40 (m/v), G2 fine-sized plant material; 5 – EtOH 70%, plant material/solvent ratio at 1:20 (m/v), G1 coarse-sized plant material; 6 – EtOH 70%, plant material/solvent ratio at 1:20 (m/v), G2 fine-sized plant material; 7 – EtOH 70%, plant material/solvent ratio at 1:40 (m/v), G1 coarse-sized plant material; 8 – EtOH 70%, plant material/solvent ratio at 1:40 (m/v), G2 fine-sized plant material; 9 – MeOH 50%, plant material/solvent ratio at 1:20 (m/v), G1 coarse-sized plant material; 10 – MeOH 50%, plant material/solvent ratio at 1:20 (m/v), G2 fine-sized plant material; 11 – MeOH 50%, plant material/solvent ratio at 1:40 (m/v), G1 coarse-sized plant material; 12 – MeOH 50%, plant material/solvent ratio at 1:40 (m/v), G2 fine-sized plant material. Values are presented as means ± SD, n = 3 per treatment group. Data without a common superscript letter differ ($P < 0.05$) as analyzed by one-way ANOVA and the TUKEY test.

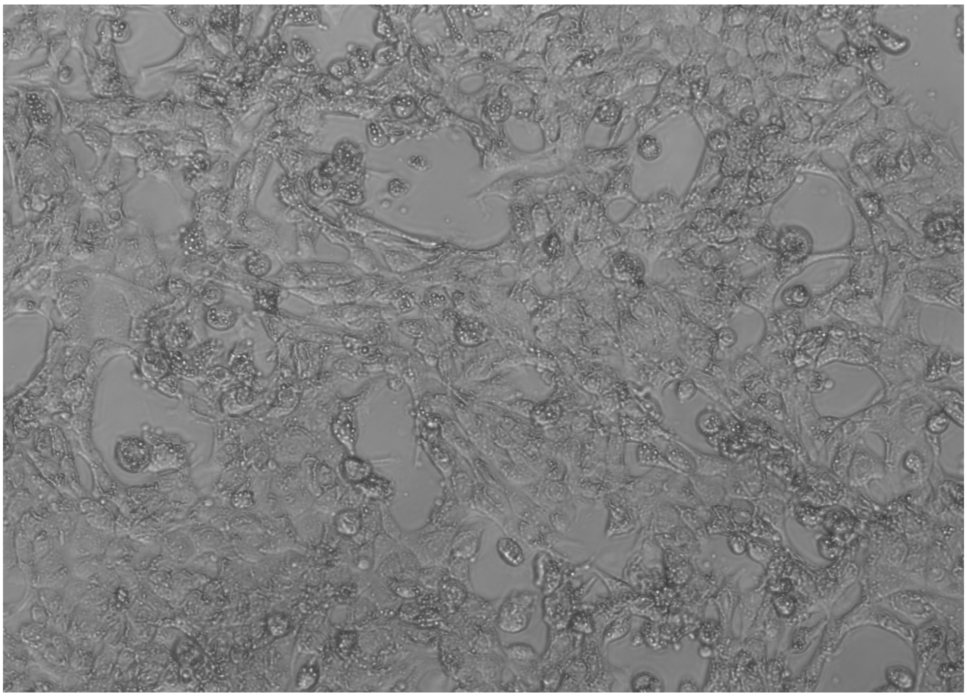

(a)

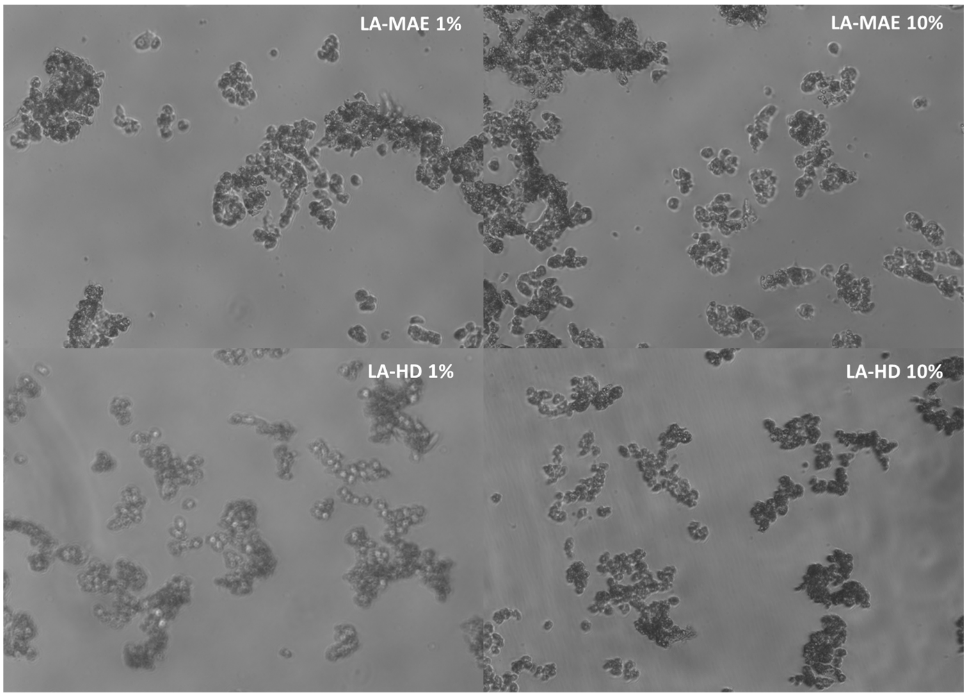

(b)

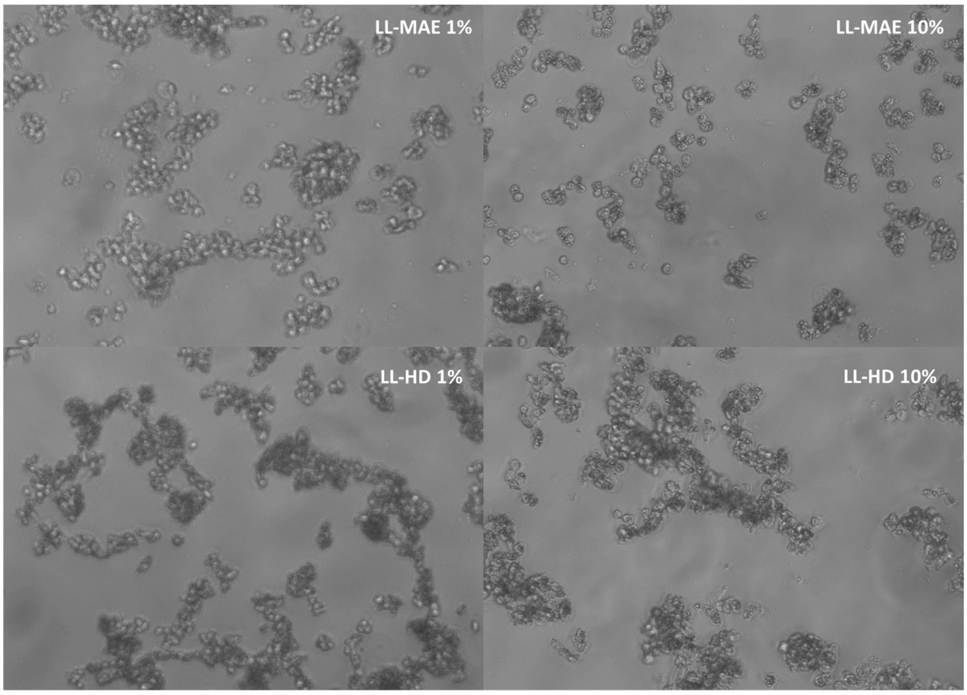

(c)

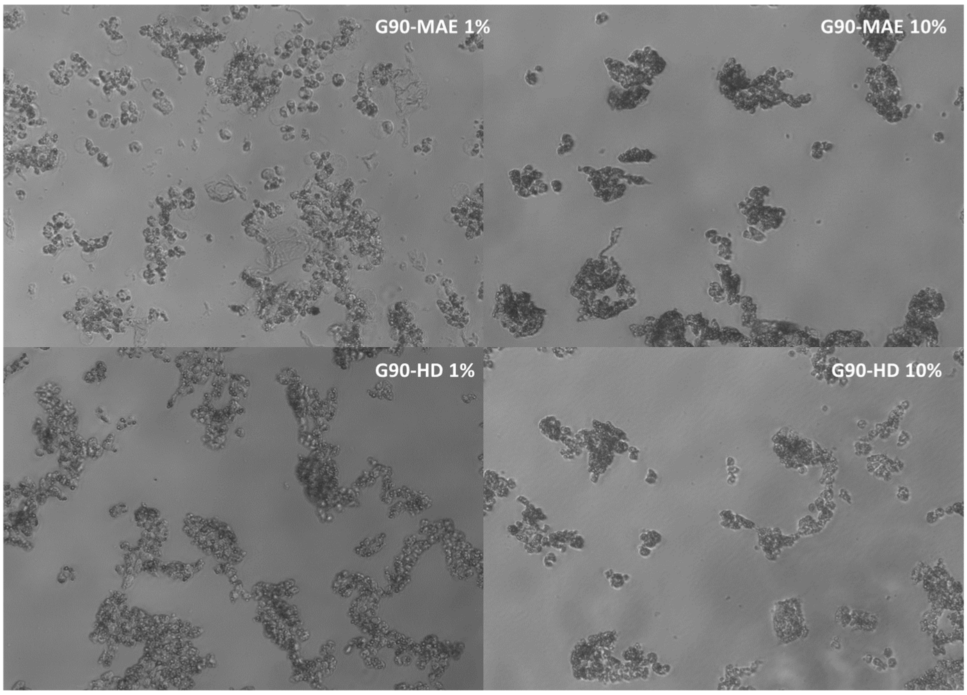

(d)

Figure S3. Cell morphological changes induced by the (a) negative control, (b) LA, (c) LL and (d) G90 essential oils.
